# Synthesis of ferrocenyl benzimidazole derivatives as novel anti-*Toxoplasma gondii* agents†

**DOI:** 10.1039/d3nj05116a

**Published:** 2024-09-10

**Authors:** Malcolm T. Ndlovu, Clare R. Harding, Catherine H. Kaschula, Prinessa Chellan

**Affiliations:** a Department of Chemistry and Polymer Science, Stellenbosch University Stellenbosch Western Cape South Africa pchellan@sun.ac.za +2721 8083327; b Wellcome Centre for Integrative Parasitology, Institute of Infection, Immunity and Inflammation, University of Glasgow Glasgow UK

## Abstract

Toxoplasmosis, a disease caused by the apicomplexan parasite *Toxoplasma gondii*, affects up to one third of the global population. Although immunocompetent individuals rarely experience severe symptoms, those with immunodeficiencies may potentially face fatal disease. The frontline treatments are currently sulphadiazine and pyrimethamine, which suffer from adverse side effects, and lack efficiency in clearing parasite cysts from the muscles and brain of patients. To address the need for novel, more effective, and less toxic treatments, four new ferrocenyl benzimidazole complexes 15–18 were synthesised and evaluated against the ΔKu80:mNeonGreen strain of *T. gondii*. Complexes 15 and 17 were found to be active with EC_50_ values of 17.9 and 17.5 μM respectively, with comparable activity to pyrimethamine, which had an EC_50_ value of 13.8 μM, and less effective than sulphadiazine, which had an EC_50_ value of 2.56 μM. Additionally, the compounds were found to be relatively non-toxic against HEK 293T and PNT1A human cell lines. Further investigations found that the complexes act by generating reactive oxygen species (ROS) through the ferrocenyl moiety. These complexes show potential for the development of new treatments against Toxoplasmosis.

## Abstract 2

New ferrocenyl-benzimidazole compounds with anti-*Toxoplasma gondii* activity were synthesized and characterized. The compounds had redox activity and led to the accumulation of reactive oxygen species within parasites.

## Graphical abstract

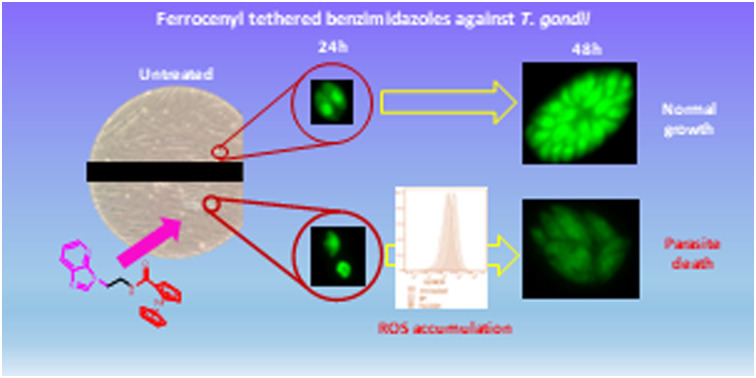

## 1. Introduction

*Toxoplasma gondii*, the causative agent of toxoplasmosis, is an obligate intracellular apicomplexan parasite. The parasite has a worldwide distribution and can infect almost all warm-blooded animals, making it one of the leading causes of reproductive loss in small livestock.^1,2^ In humans, toxoplasmosis is acquired through the consumption of undercooked meat from livestock harbouring infective growth stages of the parasite.^3^ Roughly 30% of the global population is chronically infected with *T. gondii*, making it one of the most prevalent parasitic infections,^1,4^ although, infected individuals rarely present severe symptoms, and the infection is normally resolved without the need for treatment.^5^ Immunocompromised individuals, however, may face fatal disease, with symptoms that include mental impairment, tiredness and hydrocephalus.^6–8^ The frontline treatment of toxoplasmosis involves the use of combinations of antifolate drugs such as sulphadiazine and pyrimethamine which target the active stage (tachyzoites) of the infection.^7^ Between 40 and 50% of patients receiving this treatment, however, develop severe side effects that often lead to the cessation of the therapy.^9^ Furthermore, these medications cannot efficiently eliminate parasite cysts from the muscles and brain of infected individuals.^8^ There has also been the emergence of resistance to these drugs in recent times.^8,10^ It is thus necessary to develop new, more efficacious treatments for *Toxoplasma gondii* with fewer side effects.

**Fig. 1:**
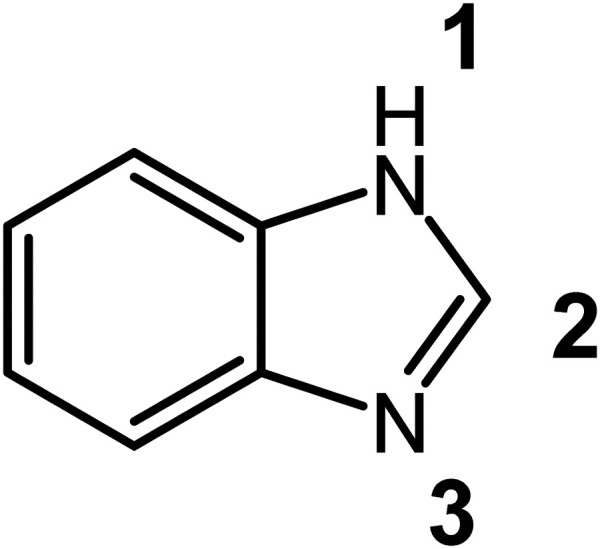
Structure of 1*H*-benzimidazole numbered to indicate the N-1 and N-3 positions.

Benzimidazole derivatives have a wide variety of biological activities, including antiplasmodial,^11,12^ antifungal,^13–15^ and anticancer activity.^16,17^ These activities arise from the presence of the benzimidazole moiety, which is a privileged scaffold, allowing it to promiscuously interact with numerous pathological targets.^18–20^ Furthermore, prominent anthelmintic drugs such as albendazole and mebendazole have the benzimidazole moiety at the core of their structure, making it an attractive building block for the synthesis of anti-*Toxoplasma gondii* compounds.^21,22^ Benzimidazoles are also amenable to derivatisation, being stable to acid and base, and resistant to reduction.^23^ Benzimidazoles undergo *N*-alkylation at the 1 and/or 3 positions (Fig. 1) to produce salts.^23,24^

Since the discovery of cisplatin, the metal containing anticancer drug, metal complexes have received considerable attention for the treatment of many diseases including toxoplasmosis. Anti-toxoplasmosis organometallic complexes bearing iron, ruthenium, zinc and cobalt metal centres amongst others have been successfully developed with good biological activity.^25–28^ The use of benzimidazole based ligands for the synthesis of biologically active organometallics has proved to be a particularly successful strategy, giving rise to compounds with anticancer^29–32^ antimalarial,^33–36^ and in the context of the current work, anti-toxoplasma activity.^37^

In this study, a linker to a ferrocenyl moiety was attached to the benzimidazoles *via N*-alkylation, the rationale being that hybridisation of bioactive organic pharmacophores with ferrocene, has been shown to produce drug hybrids with good biological activity. Examples having antimalarial, anticancer and antifungal activities have been successfully developed.^38^ Lin *et al.* have reported the synthesis of ferrocenyl albendazole derrivatives.^21^ Ferrocene was tethered to the 2-position of the benzimidazole pharmacophore *via* a linker, giving rise to derivatives with better activity than the parent compounds and with low toxicity.^21^ The ruthenocenyl counterparts of these compounds exhibited high toxicity to host cells and in most instances lower anti-toxoplasma activity, highlighting the benefits of incorporating ferrocene.^21^ When hybridised, the ferrocenyl moiety introduces novel modes of action linked to the redox reactivity of the introduced metal center.^38,39^ In addition, the ferrocenyl moiety has been shown to confer favourable physicochemical properties, such as aqueous stability, lipophilicity, non-toxicity, and aromaticity similar to phenyl.^39–41^ In this study, the strategy was to harness the fusion of benzimidazole's biological activity, together with the redox activity and physicochemical properties of ferrocene to produce a new class of drug-like compounds. The result was the synthesis of four new ferrocenyl benzimidazole conjugates which were evaluated for activity against *Toxoplasma gondii (T. gondii).*

## 2. Results and discussion

### 2.1. Chemistry

**Scheme 1:**
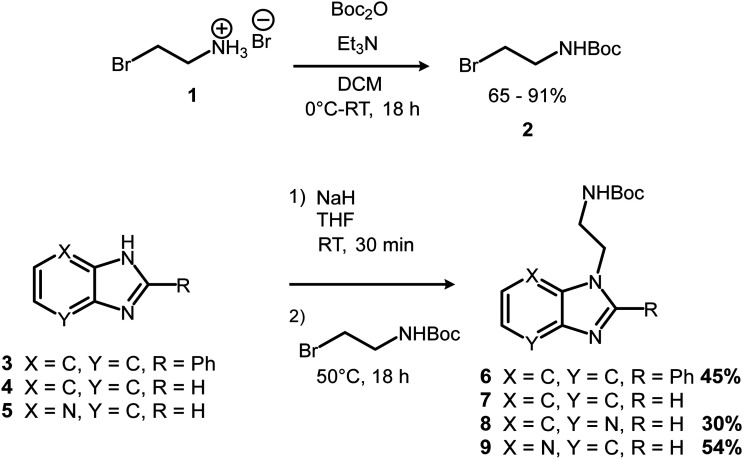
Boc-protection of bromoethylamine hydrobromide and alkylation of benzimidazoles.

**Scheme 2:**
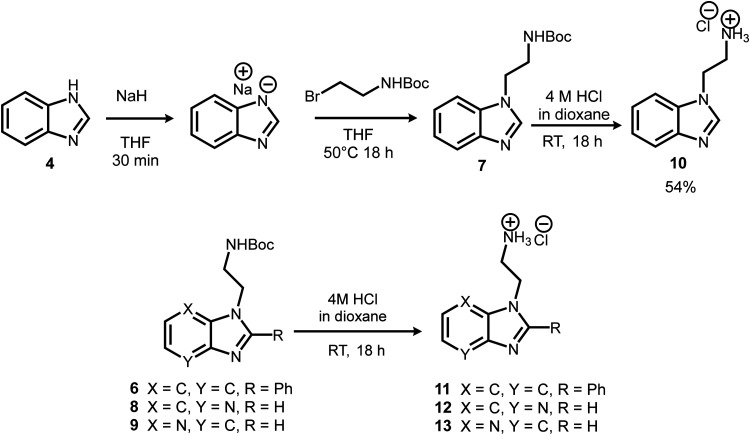
Boc-deprotection of alkyl tether.

**Scheme 3:**
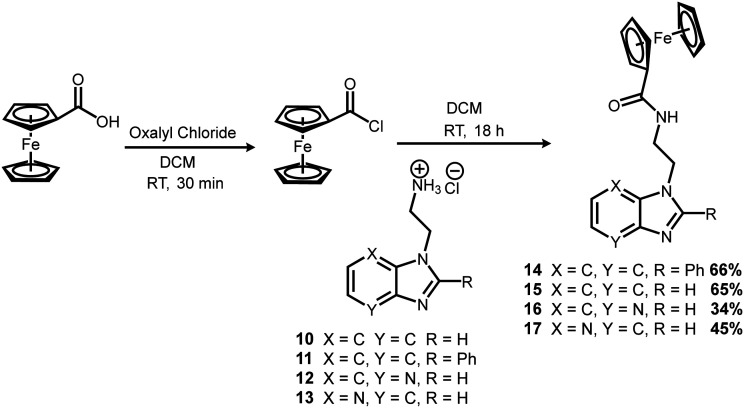
Ferrocenyl amide formation reactions to give the target compounds (14–17).

Four new ferrocenyl benzimidazole conjugates were synthesised utilising the reaction pathway shown in Schemes 1–3. Bromoethylamine hydrobromide (1) was chosen as a suitable tether to link the ferrocenyl and benzimidazole moieties, due to it having both electrophilic and nucleophilic functionalities. First, a boc-protection was carried out to prevent self-reaction, in good yield (Scheme 1). The proton nuclear magnetic resonance (^1^H NMR) spectrum of 2 matched well with that of the literature.^42^

With the protection completed, the *N*-alkylation of three commercially available benzimidazoles (3–5) could be carried out. This was done by first converting the benzimidazoles to their sodium salts using sodium hydride (NaH) as a base, followed by introduction of the alkylating agent (2) with overnight stirring at 50 °C (Scheme 1). Except for 7, the reaction products were purified and isolated using silica gel column chromatography in acceptable yields. The ^1^H NMR spectra of the products displayed two peaks each in the alkyl region between 3.40 and 4.90 ppm corresponding to the ethyl chain of the tether, as well as a characteristic singlet at approximately 1.35 ppm from the *tert*-butyl moiety of the boc-protecting group, indicating the desired products had been synthesised. Alkylation of asymmetric 4-azabenzimidazole (5) gave two regioisomer products, 8 and 9, from N1 and N3 alkylation respectively. The two regioisomers were assigned based on ^13^C NMR in which the position of alkylation results in an upfield shift in the resonance of the quaternary carbon *ipso* to it.^43^

The intermediates or pure compounds 6, 7, 8 and 9, were then boc-deprotected using 4 M HCl in dioxane (Scheme 2).^44^ The resulting products 10–13, isolated as the hydrochloride salt, were obtained in quantitative yields, and either purified by recrystallisation, or used directly without purification in the next step.

The final step involved introduction of the ferrocenyl moiety. This was done by first converting ferrocene carboxylic acid to the more reactive acyl chloride using oxalyl chloride (Scheme 3). The benzimidazole salts (10–13) were then introduced to give the final products (14–17).

All four target compounds were purified by silica gel column chromatography in poor to good yields (34–66%) and fully characterised. The ^1^H NMR spectrum of 14 contained 5 multiplet peaks in the aromatic region between 7.90 and 7.20 ppm, integrating for a total of 9 protons, accounting for the phenyl benzimidazole moiety. Peaks at 4.70 (2 protons), 4.32 (2 protons) and a prominent singlet at 4.11 (5 protons) ppm were also present, indicating the successful incorporation of the ferrocenyl moiety. The alkyl chain tethering the phenyl benzimidazole to the ferrocenyl group was still present, as indicated by the triplet at 4.50 ppm, and the multiplet at 3.75 ppm, both integrating for 2 protons each. The ^1^H NMR spectra of the other target compounds (15–17) followed the same trend, with the benzimidazole peaks appearing in the aromatic region, the ferrocenyl peaks at 4.80 to 4.00 ppm, and the two alky chain peaks each integrating for 2 protons. Overall, the significant change in the ^1^H NMR spectra of the target compounds, in comparison to their precursors, was the appearance of 3 new peaks between 3.50 and 5.00 ppm corresponding to the unsubstituted and functionalised rings of the ferrocenyl moieties. Analysis through reverse phase high performance liquid chromatography indicated that the target complexes were obtained at >90% purity. All complexes were further characterised by electrospray ionisation mass spectroscopy (positive mode). The mass spectra of 14 and 15 displayed the molecular ion ([M + H]^+^) with an abundance of 100%, and its sodium adduct ([M + Na]^+^) with 10% abundance, while the spectra of 16 and 17 displayed the molecular ion ([M + H]^+^) with an abundance of 100%.

### 2.2. Investigation of physicochemical properties of the complexes

#### 2.2.1. Turbidimetric solubility assays

The aqueous solubility of potential drug candidates is important to achieve sufficient bioavailability, and for oral delivery.^45–47^ Compounds with solubility <10 μg mL^−1^ are considered to have poor solubility, while a solubility of 10–60 μg mL^−1^ is considered moderate, and >60 μg mL^−1^ is considered good.^48^

The solubility of the synthesised target compounds 14–17, was investigated in phosphate buffered saline (PBS, pH 7.4) and in 4-(2-hydroxyethyl)-1-piperazineethanesulfonic acid (HEPES, pH 7.0) buffer, both at 37 °C using a turbidimetric solubility assay. Hydrocortisone and reserpine, drugs possessing good and poor aqueous solubility respectively, were included in the assays as controls. All the target complexes displayed poor solubility at pH 7.4, with solubility ranges between 0 and 9 μg mL^−1^ (Table S1, ESI†). At pH 7.0, 14, 16 and 17 showed poor solubility (14 : 2–4 μg mL^−1^, 16 : 4–7 μg mL^−1^17 : 0–2 μg mL^−1^), whereas 15 had moderate solubility (7–15 μg mL^−1^). Thus, the target compounds displayed poor to moderate solubility in aqueous solution at pH 7.0 and 7.4.

#### 2.2.2. Aqueous stability studies

The stability of complexes 14–17 in an aqueous environment was investigated. The stability experiments were performed in PBS (pH 7.4) and HEPES (pH 7.0) buffers, as well as in acidic acetate buffer (pH 4.6), by monitoring the UV-vis spectra of the compounds at 37 °C over an 18-hour period.

**Fig. 2:**
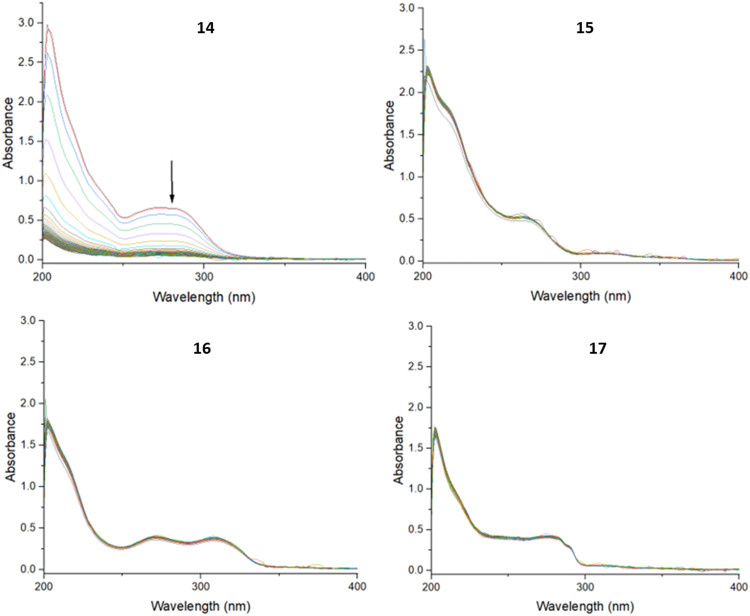
UV spectra of 14–17 in PBS buffer (pH 7.4) recorded every 5 minutes for 18 hours.

In all three buffers, the UV-spectra of compounds 15, 16 and 17 remained unchanged, indicating stability under these conditions, while the spectrum of 14 showed reduced intensity over time. An example of representative spectra can be found in Fig. 2.

To further investigate the stability of 14, the PBS, HEPES and acetate buffer solutions were evaporated to dryness, and then analysed using electrospray ionisation mass spectrometry in the positive ionisation mode. Analysis of the buffer samples of 14 showed the presence of a molecular ion peak (Fig. S50 and S51, ESI†), indicating that the decreased UV absorption in these buffers may have been due to some precipitation, but not degradation. Thus, it appears that 14 remained intact and was stable in the three aqueous environments, although possibly with reduced solubility over time.

#### 2.2.3. Cyclic voltammetry

Ferrocene is known to undergo reversible oxidation and reduction, cycling between its 3^+^ ferrocenium and the 2^+^ ferrocene (Fc) states.^49,50^ This redox behaviour leads to the generation of reactive oxygen species (ROS) through Fenton like reactions, which can in turn lead to parasite death.^51–53^ Cyclic voltammetry was used to determine if the synthesised ferrocenyl derivatives had the potential to have an impact on the redox status of the parasite cells. Two different scan rates (25 and 90 mV s^−1^) were used to ensure the reliability of the results, compared to ferrocene alone.

Ferrocene, which is known to undergo reversible oxidation and reduction was found to have Δ*E*_p_ values of 108 and 83 mV, at scan rate of 25 and 90 mV s^−1^ respectively (Table 1). Within the scan rate of −1 to 1 mV, the non-ferrocenyl-containing compounds 10–13 showed no electrochemical activity, as expected. The target compounds 14–17 bearing the ferrocenyl moiety however, displayed single electron oxidation and reduction peaks, all with Δ*E*_p_ values lower than that of ferrocene. This result indicated that the redox processes observed were electrochemically reversible.^54^

**Table 1:** Half-wave potentials (*E*_1/2_) and peak-to-peak separations (Δ*E*_p_) of the target compounds, ferrocene (Fc), and ferrocene carboxylic acid (FcCOOH) determined by cyclic voltammetry

Compound	*E*_1/2_ (mV)		Δ*E*_p_ (mV)	
	Run1 (25 mV s^−1^)	Run2 (90 mV s^−1^)	Run1 (25 mV s^−1^)	Run 2 (90 mV s^−1^)
Fc	451	453	108	83
FcCOOH	688	689	89	71
14	638	641	89	75
15	653	643	105	78
16	632	642	99	69
17	620	618	79	68

The half wave potential, *E*_1/2_ is a value which is characteristic to a specific compound. High values for *E*_1/2_ indicate a compounds resistance to electron loss.^55^ Similar values for, *E*_1/2_ were observed for all the ferrocenyl target compounds 14–17 between the two scan rates employed. These half wave potentials were higher than ferrocene, indicating they are less readily oxidised. However, the half wave potentials of all the target compounds were lower than ferrocene carboxylic acid (FcCOOH), indicating that conjugation of the ferrocenyl moiety leads to more facile oxidation. These experiments show that the target compounds undergo an electrochemically reversible single electron oxidation and reduction.

### 2.3. *In vitro* evaluation of the complexes against *Toxoplasma gondii*

#### 2.3.1. *Toxoplasma gondii* growth inhibition assay

Growth inhibition assays were carried out using the RhΔKu80:mNeonGreen strain of *T. gondii*, which expresses the mNeonGreen fluorescent protein.^56^ Parasite growth inhibition was determined by measuring the fluorescence emission of parasite growth in increasing concentrations of compounds of interest. The synthesised precursors (10–13) and the target compounds (14–17) were screened. Of all the compounds, ferrocenyl benzimidazole derivatives 15 and 17, were the only compounds bearing activity with EC_50_ values of 17.9 and 17.5 μM respectively (Table 2). The EC_50_ values of the precursors (10–13) could not be determined within the tested concentration range. The ferrocenyl stating materials, FcCOOH and its acid chloride derivative FcCOCl, were also evaluated and had no activity. Given the high structural similarity, it is not immediately clear why compound 17 was active, but its isomer, 16 was not. The key difference between the two compounds is the position of the ferrocene bearing tether relative to the nitrogen at the 4-position of the azabenzimidazole. It is therefore possible that intramolecular hydrogen bonding between this nitrogen and the amide N–H hydrogen could be taking place, keeping compound 17 in a favourable conformation, while the conformation of compound 16 renders it inactive. With regards to compound 14, from visual inspection of the structure, it bears a hydrophobic phenyl substituent at the 2-position of the benzimidazole scaffold. This, combined with the hydrophobic nature of the ferrocene functionality, may have led to the precipitation of the compound in aqueous media. During stability testing, a decline in the UV-vis spectrum of **14** was observed, which may be indicative of this precipitation (Fig. 2). The *T. gondii* frontline treatments sulfadiazine and pyrimethamine were also evaluated against the parasites as controls and were found to have EC_50_ values of 2.56 and 13.83 μM respectively. Although sulfadiazine was approximately seven times more active than the two target compounds, they had comparable activity to pyrimethamine, which is a promising result.

**Table 2:** *In vitro T. gondii* inhibition data for 15, 17 and Toxoplasmosis frontline treatments. Results represent three biological replicates performed in triplicate

Compound	EC_50_ (μM)	95% CI
14	NA^a^	NA
15	17.9	14.4–22.3
16	NA	NA
17	17.5	13.3–22.60
Sulfadiazine	2.56	1.40–7.79
Pyrimethamine	13.8	11.2–17.0

a NA: Not active at the maximum tested concentration.

#### 2.3.2. Determination of ROS generation through fluorescence activated cell sorting (FACS)

Once it had been established that two of the synthesised ferrocenyl benzimidazole derivatives were biologically active, their potential mode of action was explored. Given the presence of the ferrocenyl moiety, and the cyclic voltammetry results, it was of interest to investigate whether the compounds induce ROS, which may provide an explanation for their anti-parasitic activity. Thus, cells heavily infected with parasites were incubated with target compounds for 18 hours, before isolating the parasites and staining them with CellRox deep red reagent. CellRox is an oxidation sensitive dye which is non-fluorescent in its reduced state but fluoresces bright red at 665 nm upon being oxidised by ROS. FACS was then used to quantify CellROX parasite florescence.^56^ Parasites were incubated with ferrocene carboxylic acid (FcCOOH), and untreated parasites were used as controls.

**Fig. 3:**
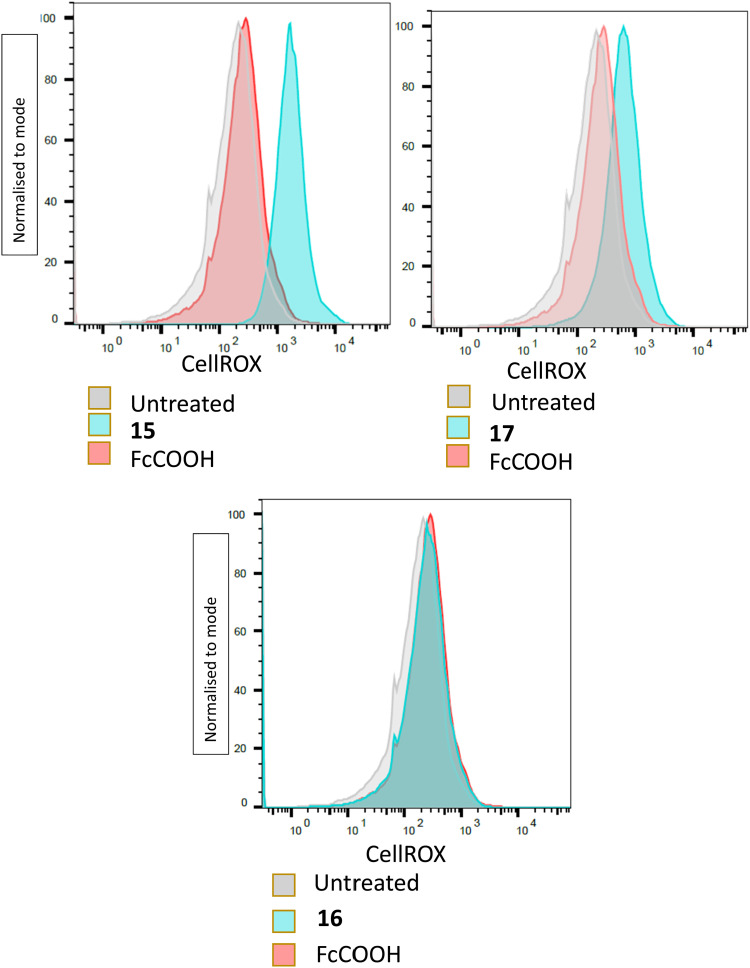
Determination of ROS formation by flow cytometry. Plots indicating parasites exhibiting red fluorescence, at least 10 000 parasites analysed/condition.

The results indicated that the compounds 15 and 17 which inhibited parasite growth, but not the inactive 16, triggered the formation of ROS within the treated parasites, with an increase red fluorescence (Fig. 3). Ferrocene itself is reported to take part in Fenton-like reactions which generate ROS,^51^ however, parasites incubated with FcCOOH, only showed slightly more ROS present than untreated parasites. This result was consistent with the cyclic voltammetry results, which showed that FcCOOH was more resistant to electron loss than the target compounds. There was however no correlation between the amount of ROS detected and the EC_50_ values of the respective compounds. Despite this, the above results showed that the active target compounds were able to affect the redox status of the parasites, leading to detectable oxidative stress. The exact mechanism by which this occurs was however not elucidated.

#### 2.3.3. *Toxoplasma gondii* growth inhibition assay in the presence of *N*-acetylcysteine (NAC)

**Fig. 4:**
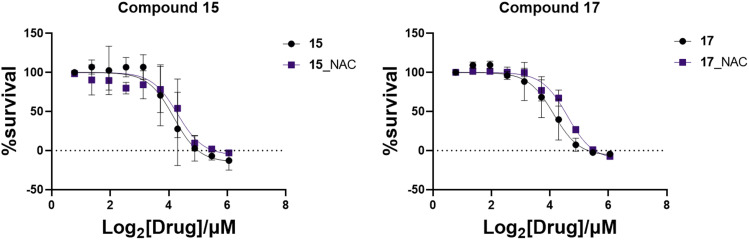
Growth inhibition curves of the active target compounds in the presence and absence of NAC. Results represent three biological replicates performed in triplicate, points at mean ± SEM.

After establishing that treatment with compounds led to ROS accumulation, growth inhibition assays in the presence of *N*-acetyl cysteine (NAC), a ROS scavenger, were carried out.^56,57^ Cells were treated with 5 mM NAC in the presence of target compounds to determine if scavenging of ROS protected parasites from toxicity. As can be seen in Fig. 4, only a slight, non-significant, shift in the parasite growth inhibition curves was observed following incubation with NAC. Thus, parasite inhibition is not solely due to ROS accumulation, suggesting another mode of action is important.

#### 2.3.4. Fluorescence microscopy

**Fig. 5:**
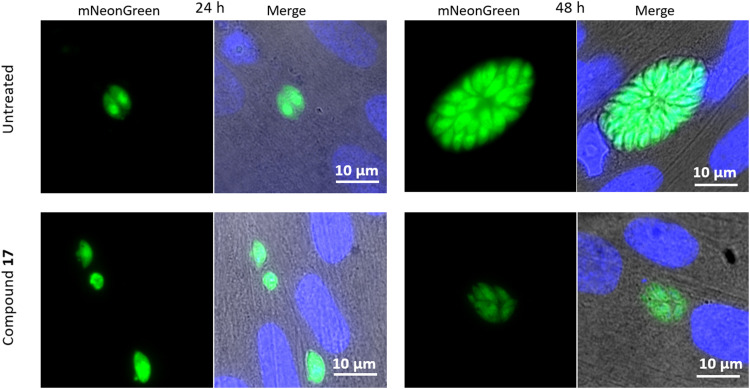
Fluorescence microscopy images of cells infected with *T. gondii*, treated with 17. Host cell nuclei shown in blue visualised with DAPI.

To visualise the impact of the target compounds on the parasites, fluorescence microscopy was performed. Compound 17 was used as a representative for the two active compounds and was incubated with infected cells at a concentration of 50 μM for 24 and 48 hours. Fluorescence microscopy images were obtained at each of the timepoints (Fig. 5).

After 24 hours, damage to the treated parasites’ outer lipid membrane was observed, as indicated by the leakage of green fluorescent protein from the parasites. At 48 hours, the parasites which had continued to proliferate, showed abnormal division of daughter cells. There were fewer parasites than the norm in the cluster observed, with evident deformation of the parasites, indicating difficulty in dividing.

#### 2.3.5. Cytotoxicity testing against human embryonic kidney 293 (HEK293T) and human prostatic (PNT1A) cell lines

**Fig. 6:**
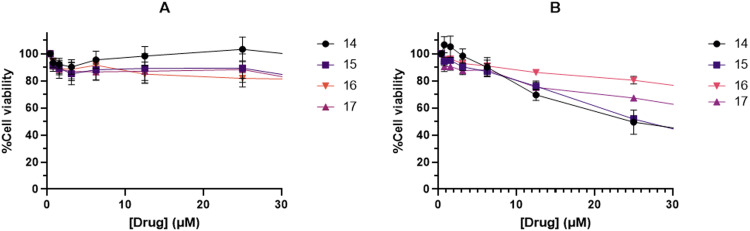
Growth inhibition curves of the target compounds against A: PNT1A and B: HEK293T cell lines. Results represent two biological replicates, each performed in triplicate, points at mean ± SEM.

The cytotoxicity of the target compounds (14–17) and their precursors (10–13) was investigated against HEK293T and PNT1A normal human cell lines using the 3-[4,5-dimethylthiazol-2-yl]-2,5-diphenyltetrazolium bromide (MTT) assay. The results showed that all the synthesised compounds were non-toxic towards the PNT1A cell line (Fig. 6A). At the maximum tested concentration (50 μM), the cells showed survival greater than or equal to 70%. For all the four compounds, the survival began to decline from 25 μM, which is higher than the EC_50_ established for 15 and 17 against *T. gondii*.

Against the HEK293T cell line, the complexes showed greater cytotoxicity (Fig. 6B). As the concentration of complex was increased, the % survival of the cells gradually declined. At the concentration of 25 μM, 15 showed a cell survival of 50%. Complex 17 proved less cytotoxic, with a % cell viability of 70% at 25 μM. Despite the cytotoxicity observed against the HEK293T cell line, the results were promising as the complexes were selective against the parasites.

## 3. Conclusion

Four new ferrocenyl benzimidazole derivatives 14–17 were successfully synthesised and characterised and their physicochemical properties were investigated. The compounds were found to have poor to moderate solubility at pH 7.0 and pH 7.4. Stability studies revealed the compounds are stable at pH 7.4, pH 7.0 and pH 4.6 although precipitation occurred over time for 14. Cyclic voltammetry revealed that the complexes underwent electrochemically reversible single electron oxidation and reduction suggesting that they have the potential to generate ROS. Compounds 15 and 17 were found to be active against *T. gondii in vitro* with EC_50_ values of 17.9 and 17. 5 μM respectively comparable to pyrimethamine, which had an EC_50_ value of 13.8 μM, and less than sulphadiazine (EC_50_ = 2.56 μM). Although the compounds were less effective than sulphadiazine, they were found to have low toxicity against normal human cell lines. Investigations into the mode of action of the ferrocenyl benzimidazole derivatives showed that the active compounds led to detectable oxidative stress in the parasites. Fluorescence microscopy imaging of parasites treated with 17 revealed parasites with damaged plasma membranes, characterised by the leakage of the fluorescent protein from the parasite. Parasites which continued to grow displayed abnormal daughter cell division in the presence of 17. The results of this study are promising and point to the potential of ferrocenyl benzimidazole derivatives as effective, non-toxic agents against *Toxoplasma gondii*.

## 4. Experimental

### 4.1. Chemistry

#### 4.1.1. Chemicals and reagents

All benzimidazole starting materials, ferrocene carboxylic acid, bromoethylamine hydrobromide di-*tert*-butyl dicarbonate, sodium hydride and oxalyl chloride were purchased from Sigma-Aldrich and used for reactions without further purification. Solvents used were all analytical grade, with dry solvents dispensed from solvent purifiers. For HPLC (High performance liquid chromatography) analysis, HPLC grade MeOH was used in combination with water from a deionised water system. Deuterated solvents were used for analysis as received without the addition of internal standards. Compounds 2, 7–10 and 13 are known compounds and were synthesised using modified previously reported procedures.^42,44,58^

#### 4.1.2. Instrumentation

Agilent 400 MHz or Bruker Ascend 600 MHz NMR spectrometers were used to collect proton and carbon spectra at room temperature. Electrospray ionisation (ESI) in positive mode on a Waters Synapt G2 mass spectrometer was used to obtain the mass spectra of all novel synthesised compounds (precursors and target compounds). Infrared spectra were recorded on a Thermo Nicolet Avatar 330 FTIR spectrometer with an attenuated total reflection (ATR) crystal. HPLC for purity testing was carried out on an Agilent 1260 Infinity II setup equipped with an autosampler. An Agilent Poroshell 120 C18 column having a pore size of 4 μm was used. A mobile phase of (A) 0.10% TFA in H_2_O/(B) 0.10% TFA in MeOH was used for analysis through gradients of *t* = 0.10% B, *t* = 15.80% B, *t* = 35.80% B, *t* = 37.10% B, *t* = 45.10% B over 45 minutes with a flow rate of 1 mL min^−1^. A detection wavelength of 230 nm was used in combination with 360 nm as the reference wavelength. 50 μL of sample dissolved in 10% MeOH/90% H_2_O were injected with a needle wash of 100% MeOH being carried out between each injection. Percentage area was obtained through the manual integration of recorded chromatograms.

#### 4.1.3. Synthesis of target compounds

##### 4.1.3.1. Bromoethylamine hydrobromide Boc protection (2)

Under N_2_ gas, an oven dried 250 mL flask was charged with Boc_2_O (1.01 g, 4.58 mmol) in dichloromethane (DCM) (5.00 mL) at 0 °C. 2-Bromoethylamine hydrobromide (1.41 g, 6.87 mmol) was added in one portion at 0 °C, followed by the dropwise addition of triethylamine (Et_3_N) (1.30 mL, 9.16 mmol) over 10 min. The reaction mixture was allowed to warm to room temperature and stirred for 18 hours. The colourless reaction mixture was diluted with DCM (15.0 mL) and washed with saturated aqueous NH_4_Cl (2 × 20.0 mL), sat. aq. NaHCO_3_ (2 × 20.0 mL), and then brine (2 × 20.0 mL). The combined organic layers were dried over anhydrous MgSO_4_, filtered, and concentrated under reduced pressure, resulting in a pale-yellow oil, 929 mg, 91%. ^1^H NMR (45 MHz, cdcl_3_) *δ* 4.93 (s, 1H), 3.58–3.44 (m, 4H), 1.45 (s, 9H).

##### 4.1.3.2. Phenyl benzimidazole alkylation (6)

Phenyl benzimidazole (0.706 g, 3.63 mmol) in THF (5.00 mL) was added to a stirring solution of NaH (0.177 g, 4.51 mmol) in THF (2.00 mL) under nitrogen at 0 °C in an ice bath. The solution was then allowed to warm up to room temperature and stirred for 30 min, resulting in a light brown precipitate. The alkylating agent dissolved in 2.00 mL THF (0.90 g, 4.00 mmol) was then added and the reaction mixture stirred overnight at 60 °C. The resulting mixture was quenched with 2.0 mL distilled water and all solvent removed under reduced pressure. The crude reaction mixture was purified by silica gel chromatography to give a light orange solid. 0.55 g, 45%. ^1^H NMR (400 MHz, cdcl_3_) *δ* 7.86–7.82 (M, 1H), 7.74–7.72 (m, 2H), 7.57–7.55 (m, 1H), 7.53–7.49 (m, 3H), 7.38–7.31 (m, 2H), 4.77 (s, 1H), 4.42 (t, *J* = 6.30 Hz, 2H), 3.49–3.44 (m, 2H), 1.35 (s, 9H) ppm. ^13^C NMR (600 MHz, CDCl_3_) *δ* 155.85, 153.44, 141.71, 135.46, 130.30, 129.55, 129.04, 123.54, 123.19, 119.59, 110.58, 79.92, 44.51, 40.10, 28.39. IR (ATR) *ν* = 3234 (N–H), 3159, 2922(C–H), 1698(C=O) cm^−1^. ESI-MS *m*/*z* 338.1883 ([M + H]^+^, 100%). Exact mass: 337.1790 g mol^−1^.

##### 4.1.3.3. Benzimidazole alkylation and deprotection (10)

Benzimidazole (0.446 g, 3.78 mmol) in THF (5.00 mL) was added to a stirring solution of NaH (0.166 g, 4.15 mmol) in THF (2.00 mL) under nitrogen at 0 °C in an ice bath. The solution was then allowed to warm up to room temperature and stirred for 30 min, resulting in an off-white precipitate within a yellowish solution. The alkylating agent (0.930 g, 4.15 mmol) dissolved in 2.00 mL THF was then added and the reaction mixture stirred overnight at 50 °C. The resulting mixture was quenched with 2.00 mL distilled water and all solvent removed under reduced pressure. The resulting light-yellow crude product was dissolved in 4.00 mL dioxane and 2.55 mL of 4 M HCl in dioxane (11.3 mmol) were added to the stirring solution. This was then stirred overnight (18 hours) at RT, resulting in a white solid. The crude reaction product was purified by recrystallisation from MeOH and EtOAc to give a white powder. Yield: 0.351 g, 54%. ^1^H NMR (400 MHz, d_2_o) *δ* 9.40 (s, 1H), 7.96–7.90 (m, 2H), 7.76–7.70 (m, 2H), 4.96 (t, *J* = 6.40 Hz, 2H,), 3.72 (t, *J* = 6.40 Hz, 2H) ppm. ^13^C NMR (600 MHz, D_2_O) *δ* 141.67, 132.04, 127.87, 127.67, 116.01, 113.01, 44.62, 38.74. IR (ATR) *ν* = 3106(N–H) 2733(C–H) 1612(N–H) cm^−1^. ESI-MS *m*/*z* 162.1036 ([M − Cl]^+^, 100%). Exact mass: 197.0720 g mol^−1^.

##### 4.1.3.4. 4-Azabenzimidazole alkylation (8 & 9)

4-Azabenzimidazole (0.488 g, 4.09 mmol) in DMF (5.00 mL) was added to a stirring solution of NaH (0.177 g, 4.51 mmol) in DMF (2.00 mL) under nitrogen at 0 °C in an ice bath. The solution was then allowed to warm up to room temperature and stirred for 30 min, resulting in a light brown precipitate. The alkylating agent dissolved in 2.00 mL DMF (1.09 g, 4.51 mmol) was then added and the reaction mixture stirred overnight at 60 °C. The resulting mixture was quenched with 2.0 mL distilled water and all solvent removed under reduced pressure. The crude reaction mixture was purified by silica gel chromatography, leading to the separation of the starting material from two alkylation products.

N-3 isomer (9): 0.572 g, 54%. ^1^H NMR (400 MHz, acetone) *δ* 8.35 (dd, *J* = 4.70, *J* = 1.30 Hz, 1H), 8.23 (s, 1H), 8.00–7.98 (m, 1H), 7.25 (dd, *J* = 7.90, *J* = 4.70 Hz, 1H), 6.27 (s, 1H), 4.45 (t, *J* = 5.80 Hz, 2H), 3.62–3.54 (m, 2H), 1.35 (s, 9H) ppm. ^13^C NMR (400 MHz, cdcl_3_) *δ* 155.41, 146.65, 145.21, 144.33, 134.28, 125.76, 118.95, 83.32, 46.22, 41.60, 28.40. IR (ATR) *ν* = 3346.88 (N–H), 2975.18 (C–H), 1676.66 (C=O) cm^−1^. ESI-MS *m*/*z* 263.1520 ([M + H]^+^, 100%). Exact mass: 262.1430 g mol^−1^.

N-1 isomer (8): 0.324 g, 30%. ^1^H NMR (400 MHz, acetone) *δ* 8.42 (dd, *J* = 4.70, *J* = 1.60 Hz, 1H), 8.26 (s, 1H,), 7.98 (dd, *J* = 8.10, *J* = 1.60 Hz, 1H), 7.24 (dd, *J* = 8.10, *J* = 4.70 Hz, 1H), 6.37 (s, 1H), 4.46 (t, *J* = 5.80 Hz, 2H), 3.57–3.52 (m, 2H), 1.35 (s, 9H) ppm. ^13^C NMR (400 MHz, cdcl_3_) *δ* 156.81, 156.23, 151.46, 139.69, 133.50, 130.12, 114.77, 80.19, 54.40, 40.01, 27.91. IR (ATR) *ν* = 3410.96 (N–H), 2964.34 (C–H), 1685.20 (C=O) cm^−1^. ESI-MS *m*/*z* 263.1520 ([M + H]^+^, 100%). Exact mass: 262.1430 g mol^−1^.

##### 4.1.3.5. Boc-deprotection of alkylated phenyl benzimidazole (11)

*tert*-Butyl (2-(2-phenyl-1*H*-benzo[*d*]imidazol-1-yl)ethyl)carbamate (0.200 g, 0.593 mmol) was placed in a round bottom flask with dioxane (4.00 mL) and the solution stirred. A prepared (4 M) solution of hydrochloric acid (HCl) in dioxane (0.445 mL, 1.78 mmol) was then added and the reaction stirred at RT overnight (18 hours). Upon completion of the reaction the solvent was removed *in vacuo*, giving product as a white powder in quantitative yields (100%), which was used for the next step without purification. ^1^H NMR (600 MHz, D_2_O) *δ* 7.96–7.94 (m, 1H), 7.91–7.85 (m, 4H), 7.81–7.78 (m, 2H), 7.76–7.71 (m, 2H), 4.94 (t, *J* = 7.14 Hz, 2H), 3.52 (t, *J* = 7.00 Hz, 2H) ppm. ^13^C NMR (600 MHz, D_2_O) *δ* 151.52, 134.22, 132.55, 131.38, 130.64, 130.26, 127.92, 127.57, 122.20, 115.36, 113.00, 42.95, 38.05. IR (ATR) *ν* = 3401.25 (N–H), 2799.05 (C–H), 1605.02 (C=N) cm^−1^

##### 4.1.3.6. Boc-deprotection of alkylated 4-azabenzimidazoles (12 & 13)

Alkylated 4-azabenzimidazole (0.250 g, 0.953 mmol) was placed in a round bottom flask with dioxane (4.00 mL) and the solution stirred. A prepared (4 M) solution of hydrochloric acid in dioxane (0.715 mL, 2.86 mmol) was then added and the reaction stirred at RT overnight (18 hours). Upon completion of the reaction the solvent was removed *in vacuo*, giving product as a white powder in quantitative yields, which was used for the next step without purification.

N-3 isomer (13): ^1^H NMR (600 MHz, D_2_O) *δ* 9.54 (s, 1H), 8.76 (d, *J =* 4.80 Hz, 1H), 8.40 (d, *J* = 8.30 Hz, 1H), 7.78–7.76 (m, 1H), 4.98 (t, *J* = 5.80 Hz, 2H), 3.75 (t, *J* = 5.70 Hz, 2H) ppm. ^13^C NMR (600 MHz, D_2_O) *δ* 148.91, 143.79, 143.37, 126.32, 125.53, 123.53, 43.99, 39.07 ppm. IR (ATR) *ν* = 3394.99 (N–H), 2848.21 (C–H) cm^−1^. ESI-MS *m*/*z* 163.0092 ([M−Cl]^+^, 100%). Exact mass: 198.0672 g mol^−1^.

N-1 isomer (12): ^1^H NMR (600 MHz, D_2_O) *δ* 8.91 (s, 1H), 8.83–8.81 (m, 2H), 7.93–7.91 (m, 1H), 5.30 (t, *J* = 6.10 Hz, 2H), 3.83 (t, *J* = 6.10 Hz, 2H) ppm. ^13^C NMR (600 MHz, D_2_O) *δ* 150.42, 148.87, 139.49, 131.81, 131.49, 120.60, 52.89, 39.24 ppm. IR (ATR) *ν* = 3445.25 (N–H), 2797.66 (C–H) cm^−1^. ESI-MS *m*/*z* 163.0993 ([M−Cl]^+^, 100%). Exact mass: 198.0672 g mol^−1^.

##### 4.1.3.7. Phenyl benzimidazole ferrocenyl conjugate synthesis (14)

To a stirred suspension of ferrocene carboxylic acid (0.126 g, 0.548 mmol) in DCM (5 mL) at RT under N_2_ gas, was added oxalyl chloride (0.548 mL, 1.09 mmol). Formation of gas was observed and within 10 minutes a dark red homogenous solution had formed. The reaction was stirred for an additional 1 hour to ensure completion of the reaction, followed by the removal of the solvent under reduced pressure to give a dark red oil. The red oil was then redissolved in fresh dry DCM (5.00 mL) and stirred at 0 °C. 2-(2-phenyl-1*H*-benzo[*d*]imidazol-1-yl)ethanaminium chloride (0.100 g, 0.365 mmol) was then dissolved in 5.00 mL DCM with Et_3_N (0.160 mL, 1.09 mmol) and the resulting solution added to the stirring acid chloride *via* syringe, releasing a white gas. When addition was complete the reaction was allowed to warm up to RT and stir for 18 hours. Upon completion, the reaction was diluted with 20.0 mL DCM and washed with 2 × 30.0 mL brine. The organic layer was dried over anhydrous MgSO_4_, filtered, and concentrated under reduced pressure. The product was purified by silica gel column chromatography using an eluent of 1 : 1 EtOAc : Hexane. Yield: 0.073 g, 64% orange solid. ^1^H NMR (400 MHz, acetone) *δ* 7.88–7.85 (m, 2H), 7.81–7.79 (m, 1H), 7.70–7.68 (m, 1H), 7.56–7.53 (m, 3H), 7.35–7.35 (m, 2H), 4.70–4.69 (m, 2H), 4.50 (t, *J* = 7.20 Hz, 2H), 4.33–4.32 (m, 2H), 4.11 (s, 5H), 3.78–3.72 (m, 2H) ppm. ^13^C NMR (600 MHz, Acetone) *δ* 170.84, 154.23, 144.25, 137.20, 131.83, 130.37, 130.33, 129.47, 123.37, 122.89, 120.39, 111.62, 77.38, 70.89, 70.34, 69.08, 44.80, 39.56 ppm. IR (ATR) *ν* = 3221 (NH), 2919, 2850 (CH), 1713 (C=O) cm^−1^. ESI-MS *m*/*z* 450.1271 ([M + H]^+^, 100%), 472.1088 ([M + Na]^+^, 10%). Exact mass: 449.1191 g mol^−1^.

##### 4.1.3.8. Benzimidazole ferrocenyl conjugate synthesis (15)

To a stirred suspension of ferrocene carboxylic acid (0.073 g, 0.318 mmol) in DCM (5.00 mL) at room temperature under Nitrogen, was added oxalyl chloride (0.318 mL, 0.636 mmol). Formation of gas was observed and within 10 minutes a dark red homogenous solution had formed. The reaction was stirred for an additional 1 hour to ensure completion of the reaction, followed by the removal of the solvent under reduced pressure to give a dark red oil. The red oil was then redissolved in 5.00 mL of fresh dry DCM and stirred at 0 °C. 2-(1*H*-benzo[*d*]imidazol-1-yl)ethanaminium chloride (0.042 g, 0.212 mmol) was then dissolved in 5.00 mL DCM with Et_3_N (0.100 mL, 0.637 mmol) and the resulting solution added to the stirring acid chloride *via* syringe, releasing a white gas. When addition was complete the reaction was allowed to warm up to RT and stirred for 18 hours. Upon completion, the reaction was diluted with 20.0 mL DCM and washed with 2 × 30.0 mL Brine. The organic layer was dried over anhydrous MgSO_4_, filtered, and concentrated under reduced pressure. The product was purified by silica gel column chromatography using an eluent of 100% EtOAc. Yield: 0.051 g, 65% orange solid. ^1^H NMR (400 MHz, acetone) *δ* 8.09 (s, 1H), 7.67–7.65 (m, 2H), 7.43 (s, 1H), 7.29–7.19 (m, 2H), 4.73–4.70 (m, 2H), 4.52–4.48 (m, 2H), 4.33–4.30 (m, 2H), 4.13–4.10 (m, 5H), 3.80–3.74 (m, 2H) ppm. ^13^C NMR (400 MHz, acetone) *δ* 170.86, 144.85, 135.19, 130.55, 123.38, 122.53, 120.69, 111.12, 77.58, 70.85, 70.31, 69.06, 45.01, 39.94 ppm. IR (ATR) *ν* = 3265(N–H), 2923(C–H), 1737(C=O) cm^−1^. ESI-MS *m*/*z* 374.0598 ([M + H]^+^, 100%) 396.0773 ([M + Na]^+^, 10%). Exact mass: 373.0878 g mol^−1^.

##### 4.1.3.9. 4-Azabenzimidazole ferrocenyl conjugate synthesis (16 & 17)

To a stirred suspension of ferrocene carboxylic acid (0.174 g, 0.756 mmol) in DCM (5.00 mL) at room temperature under nitrogen, was added oxalyl chloride (0.754 mL, 1.51 mmol). Formation of HCl gas was observed and within 10 minutes a dark red homogenous solution had formed. The reaction was stirred for an additional 1 hour to ensure completion of the reaction, followed by the removal of the solvent under reduced pressure to give a dark red oil. The red oil was then redissolved in 5.00 mL dry DCM and stirred at 0 °C. Each of the azabenzimidazole salts (0.100 g, 0.504 mmol) was then dissolved in 5.00 mL DCM with Et_3_N (0.210 mL, 1.51 mmol) and the resulting solution added to the stirring acid chloride *via* syringe, releasing a white gas. When addition was complete the reaction was allowed to warm up to RT and stirred for 18 h. Upon completion, the reaction was diluted with 20.0 mL DCM and washed with 2 × 30.0 mL Brine. The organic layer was dried over anhydrous MgSO_4_, filtered, and concentrated under reduced pressure. The product was purified by silica gel column chromatography using an eluent of 1 : 10 methanol : EtOAc.

N-3 isomer (17): 0.064 g, 34%. ^1^H NMR (400 MHz, dmso) *δ* 8.48 (s, 1H), 8.40 (dd, *J* = 4.70, *J* = 1.50 Hz, 1H), 8.15 (dd, *J* = 8.10, *J* = 1.60 Hz, 1H), 8.04–8.01 (m, 1H), 7.31–7.27 (m, 1H), 4.69–4.68 (m, 2H), 4.47–4.44 (m, 2H), 4.31–4.30 (m, 2H), 4.02 (s, 5H), 3.66–3.60 (m, 2H) ppm. ^13^C NMR (600 MHz, Acetone) *δ* 170.51, 146.10, 144.58, 136.60, 128.26, 118.78, 77.66, 70.81, 70.28, 69.01, 44.10, 40.03 ppm. IR (ATR) *ν* = 3260.13 (N–H), 2920.63 (C–H), 1626.24 (C=O) cm^−1^. ESI-MS *m*/*z* 375.0920 ([M + H]^+^, 100%). Exact mass: 374.0830 g mol^−1^.

N-1 isomer (16): 0.084 g, 45%. ^1^H NMR (400 MHz, CD_3_OD) *δ* 8.39–8.37 (m, 2H), 8.24–8.23 (m, 1H), 7.38–7.35 (m, 1H), 4.95–4.92 (m, 2H), 4.67–4.66 (m, 2H), 4.36–4.35 (m, 2H), 4.07 (s, 5H), 3.98 (t, *J* = 5.82 Hz, 2H) ppm. ^13^C NMR (400 MHz, CD_3_OD) *δ* 174.11, 159.28, 152.85, 143.35, 134.89, 131.44, 115.28, 76.11, 71.80, 70.75, 69.35, 55.00, 39.73 ppm. ESI-MS *m*/*z* 375.0894 ([M + H]^+^, 100%). Exact mass: 374.0830 g mol^−1^.

### 4.2. Investigation of physicochemical properties

#### 4.2.1. Instrumentation

HPLC for analysis of stability studies solutions was carried out on an Agilent 1260 Infinity II setup equipped with an autosampler. An Agilent Poroshell 120 C18 column having a pore size of 4 μm was used. A mobile phase of (A) 0.10% TFA in H_2_O/(B) 0.10% TFA in MeOH was used for analysis through gradients of *t* = 0.10% B, *t* = 15.80% B, *t* = 35.80% B, *t* = 37.10% B, *t* = 45.10% B over 45 minutes with a flow rate of 1 mL min^−1^. A detection wavelength of 230 nm was used in combination with 360 nm as the reference wavelength. 50 μL of sample dissolved in 5% MeOH/95% buffer (PBS, HEPES or acetate buffer) were injected with a needle wash of 100% MeOH being carried out between each injection.

#### 4.2.2. Turbidimetric assay

##### 4.2.2.1 Preparation of buffers

A single phosphate buffered saline (PBS) tablet (Sigma Aldrich) was dissolved in 200 mL of distilled water (dH_2_O) (25 °C) to obtain a PBS solution with 0.01 M phosphate buffer, 0.14 M NaCl and 0.003 M KCl. A 4-(2-hydroxyethyl)-1-piperazineethanesulfonic acid (HEPES) buffer having a concentration of 0.0025 M was prepared by dissolving 1.19 g HEPES acid (5.00 mmol) in 180 mL of dH_2_O followed by titrating the solution to a pH of 7.00 with 0.1 M NaOH. The solution was then made up to a volume of 200 mL with dH_2_O. Both buffers were filtered through syringe filters before use.

##### 4.2.2.2. Preparation of 96 well plates

Stock solutions of the compounds to be tested were prepared in DMSO at a concentration of 10 mM and filtered through syringe filters. Serial dilution of the stock solutions with DMSO was then carried out in a preparatory (prep) 96-well plate giving concentrations between 0.00 and 10.0 mM (0.00, 0.25, 0.50, 1.0, 2.0, 4.0, 8.0 and 10 mM). The solutions were then further diluted into 96-well assay plates containing DMSO, PBS or HEPES buffer by pipetting 4.00 μL of solution from the prep plate to the Assay plate containing 196 μL of DMSO or buffer, giving final concentrations between 0.00 and 200 μM (0.00, 5.00, 10.0, 20.0, 40.0, 80.0, 160 and 200 μM). The assay plates were incubated at 25.0 or 37.0 °C for two hours before their UV-vis absorbance was read at 620 nm with a SPECTROstar Nano microplate reader spectrophotometer. Blank readings were used to gain corrected readings by subtracting them from obtained absorbances for each compound concentration.

#### 4.2.3. Aqueous stability studies

##### 4.2.3.1. Preparation of buffers

PBS and HEPES buffers were prepared as described above. An acetate buffer with a concentration of 0.0065 M and a pH of 4.60 was prepared by dissolving 5.40 g (65.83 mmol) of sodium acetate in 50 mL of dH_2_O. The resulting solution was then titrated to a pH of 4.60 with glacial acetic acid and made up to 100 mL with dH_2_O.

##### 4.2.3.2. Determination of stability

1 mM stock solutions of the synthesised target compounds in MeOH were prepared. 1.90 mL of buffer (PBS, HEPES or acetate buffer) was pipetted into cuvettes followed by the addition 100 μL of test compound stock solutions, diluting them to a final concentration of 50 μM. The cuvettes were then stoppered, inverted to mix the solution, and placed in an Agilent Cary 3500 UV-vis spectrophotometer as fast as possible. Blank solutions in matched cuvettes were also prepared by adding 100 μL of MeOH to 1.9 mL of buffer. The solutions in all the cuvettes were stirred at 400 rpm ensure proper mixing. UV spectra were then recoded every 5 minutes for 18 hours with a spectral width of 200–1100 nm to monitor the stability of the test compounds.

#### 4.2.4. *T. gondii* growth inhibition assay

##### 4.2.4.1. Parasite and host cell maintenance

*T. gondii* ΔKu80:mNeon tachyzoites were grown in human foreskin fibroblasts (HFFs) with Dulbecco's modified Eagle's medium (DMEM) supplemented with 3% heat inactivated foetal bovine serum (D3) as the culturing medium. Cells and parasites were maintained at 37 °C and 5% carbon dioxide (CO_2_).

##### 4.2.4.2. Infection of host cells

EC_50_s were calculated as previously described.^56^ Briefly, *T. gondii* ΔKu80:mNeon parasites were counted by haemocytometer and diluted to 25 000 parasites per mL in 40 mL of D3 media, to give an infection of 5000 parasites per well. The media from a black clear bottomed 96 well plate containing HFFs was then aspirated. 200 μL of uninfected D3 media was added to the top and bottom rows of wells (A and H) to serve as blanks. 200 μL of *T. gondii* containing D3 media was then added to the remaining wells. The parasites were allowed 2 hours to invade host cells, after which test compounds were added.

##### 4.2.4.3. Supplementation of media with drugs

100 mM stock solutions of each of the drugs in DMSO were prepared and diluted in D3 to give a final concentration of 100 μM for each of the drugs. The media from the infected 96 well plates was then aspirated and 100 μL of D3 media added to each well, excluding column 12. The first 4 wells of column 12 were filled with 300 μL of drug supplemented D3 media, while the next 4 wells were filled with 300 μL of D3 media supplemented with a different drug. The drug supplemented D3 media was then serially diluted 1 : 3 across the plate, stopping at column 2 and disposing of the media, leaving all wells with 100 μL of media. Finally, the plates were closed and incubated at 37 °C with 5% CO_2_ for 4 days. After 4 days the fluorescence of each well on the plates was read at 594 nm emission using a PHERAstar FS microplate reader. Graphpad Prism 9 software was used to plot dose–response and determine inhibition of parasite growth.

#### 4.2.5. Determination of ROS formation through fluorescence activated cell sorting (FACS)

6 cm dishes of host cells heavily infected with *T. gondii* were untreated or treated with 66 μM (second highest concentration used in growth inhibition assays) of test compounds for 18 hours. The dishes were then scratched and syringed, before filtering the solution into 10 mL falcon tubes to remove cell debris. The tubes were spun down for 10 minutes at 1500 rpm. The media was then removed from the tubes, and the pellet resuspended in 200 μL of PBS buffer. For each drug, 100 μL of the resuspended pellet was then placed into 2 FACS tubes, one which would then be stained with CellROX dye, and the other 100 μL of PBS added to act as an unstained control. A 1 : 125 dilution of CellROX stock solution was performed, and 100 μL of this solution added to the FACS tubes needing staining. Stained tubes were incubated at 37 °C for at least 15 min before analysis on a BD Celesta flow cytometer. Data was obtained using FACSDiva software with parasites being gated on forward scatter, side scatter and green fluorescence. FlowJo v10 software was used to plot the geometric mean of CellRox fluorescence from at least 5000 parasites on the Cy7 channel.

#### 4.2.6. *T. gondii* growth inhibition assays with *n*-acetyl cystine (NAC)

Cells were infected as above. 100 mM stock solutions of each of the drugs in DMSO were prepared. 1 mL of D3 media was then supplemented with 1 μL of drug and 4 μL of NAC, to give a final concentration of 100 μM for each of the drugs, and 5 mM NAC for each test solution. The cells were treated and results quantified as described above.

#### 4.2.7. Immunofluorescence assay (IFA) (fluorescence microscopy)

A 24 well plate of HFF host cells grown on cover slips was infected with 100 μL of parasites per well and incubated at 37 °C for 1 h. The media in the wells was aspirated and replaced with media containing 50 μM of 17 The plate was once again incubated for 24 or 48 h at 37 °C. Once incubation was complete, the cells were washed once with PBS, before fixation with 500 μL of 4% paraformaldehyde (PFA) at room temperature for 20 minutes. Cells were washed and 500 μL of blocking buffer (2% bovine serum albumin (BSA) in mL PBST (PBS, 0.2% Triton x-100 solution) and incubated for 20 min at room temperature. The cover slips were then carefully removed from each well, mounted using Fluoromount with DAPI (Southern Biotech), and placed facing down on a microscope slide. Images were obtained using a Leica DiM8 (Leica Microsystems) microscope and processed and deconvolved using SoftWoRx and FIJI software.

#### 4.2.8. Cell viability (MTT assay)

##### 4.2.8.1. Preparation of MTT (3-[4,5-dimethylthiazol-2-yl]-2,5-diphenyltetrazolium bromide) solution

A 5.00 mg mL^−1^ solution of MTT in sterile PBS was prepared by weighing out 100 mg of MTT into a 50 mL Falcon tube. 20 mL of autoclaved PBS was then added to the tube before vortexing and incubating the resulting solution in a 37 °C water bath for 15 minutes. After incubation the Falcon tube was wrapped in foil and the MTT solution refrigerated and stored at 4 °C.

##### 4.2.8.2. Preparation of solubilisation reagent

A 10% solution of SDS in 0.01 M HCl was prepared by placing 50 g of SDS and 5 mL of 1 M HCl in a 500 mL Schott bottle. The volume of the solution was then made up to 500 mL with dH_2_O.

##### 4.2.8.3. HEK293T and PNT1A cell line maintenance

Human embryonic kidney 293 (HEK293T) cells were cultured in DMEM supplemented with 10% heat inactivated foetal bovine serum (FBS). Human prostatic epithelial cells (PNT1A) were cultured in Rosewell Park Memorial Institute (RPMI) media supplemented with 10% heat inactivated FBS. Both cell lines were incubated at 37 °C with 5% CO_2_. Both cell lines were obtained from ATCC.

##### 4.2.8.4. MTT assay

The cells were cultured until 75% confluency and then plated at 3000 cells per well in a 96 well plate in 90 μL of cell containing media. The cells were allowed to recover overnight before the addition of compounds.

Stock solutions of the test compounds in DMSO were prepared and serially diluted in DMSO in a 96 well plate. The compounds were further diluted by adding 2 μL of compound solution from the serial dilution plate to a 96 well plate containing 198 μL of media to give a 100 times dilution of the test compounds. 10 μL of the test compounds in media was then pipetted into the plates containing cells in triplicate. The cells were then incubated with the test compounds for 48 hours. After incubation, 10 μL of previously prepared MTT reagent was added to each well followed by incubation of the plates for 4 hours. 100 μL of solubilising agent was then pipetted into each well, and the plates incubated overnight. After overnight incubation the plates were read using a BioTek SYNERGY HTX multimode plate reader at 595 nm. Graphpad Prism 9 software was used to plot dose–response and determine cell viability.

## Data availability

The data supporting this article have been included as part of the ESI.†

## Conflicts of interest

The authors declare that they have no competing financial interests or personal relationships that could influence the work reported in this paper.

## Supplementary Material

NJ-048-D3NJ05116A-s001
