# Supplementary material for: Synthesis of ferrocenyl benzimidazole derivatives as novel anti-Toxoplasma gondii agents
Source: New J Chem. 2024 Sep 10;48(37):16415–28. doi: 10.1039/d3nj05116a (PMC11385693; doi:10.1039/d3nj05116a)
Supplement: NJ-048-D3NJ05116A-s001 [file NJ-048-D3NJ05116A-s001.pdf]

Supplementary Information (SI) for New Journal of Chemistry.
This journal is © The Royal Society of Chemistry and the Centre National de la Recherche Scientifique 2024

Electronic Supplementary information

Synthesis of ferrocenyl benzimidazole derivatives and their evaluation as anti-*Toxoplasma gondii* agents

Malcolm T. Ndlovu,[a] Clare R. Harding,[b] Prinessa Chellan,[a*] Catherine H. Kaschula.[a]

[a] Department of Chemistry and Polymer Science, Stellenbosch University, Stellenbosch, Western Cape, South Africa.

[2]Wellcome Centre for Integrative Parasitology, Institute of Infection, Immunity and Inflammation, University of Glasgow, Glasgow, UK

**Corresponding author:* *pchellan@sun.ac.za, +2721 8083327*

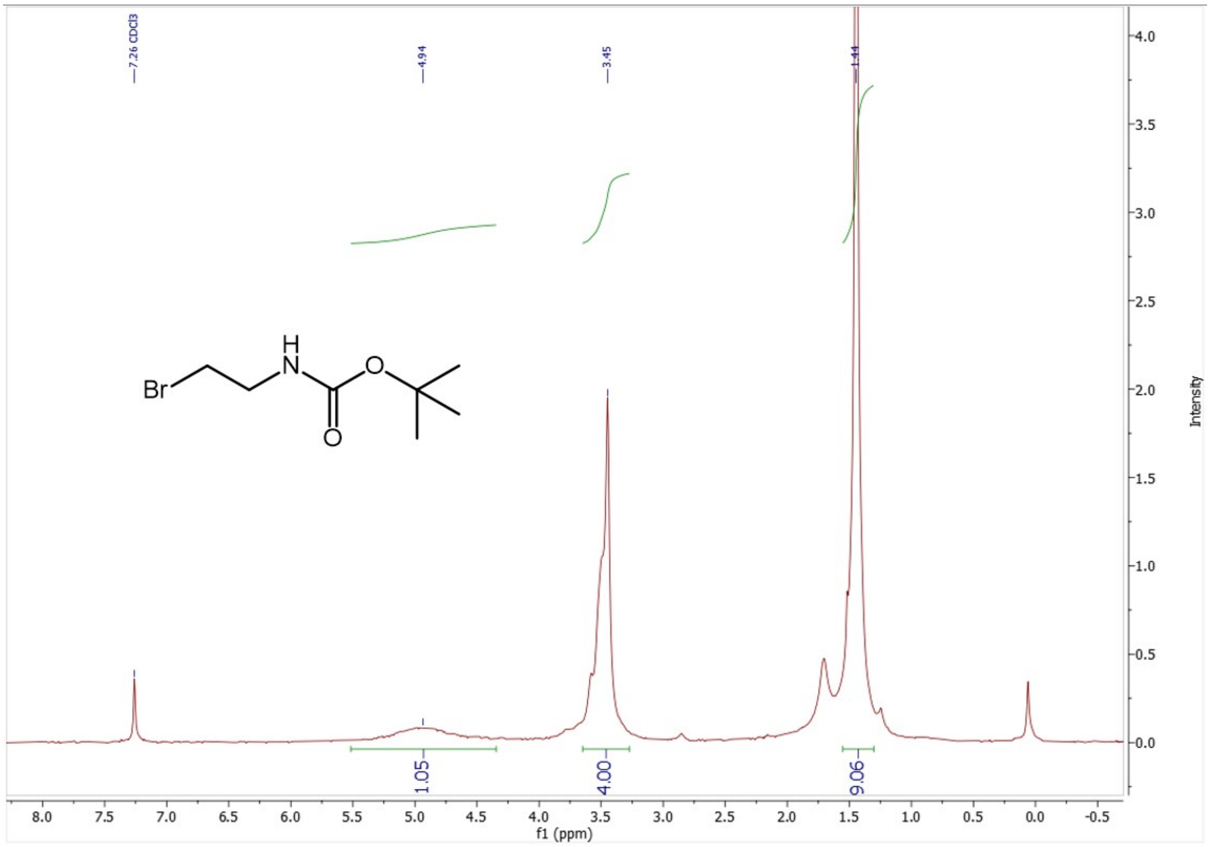

Figure S1: ^{1}H NMR spectrum of **2**

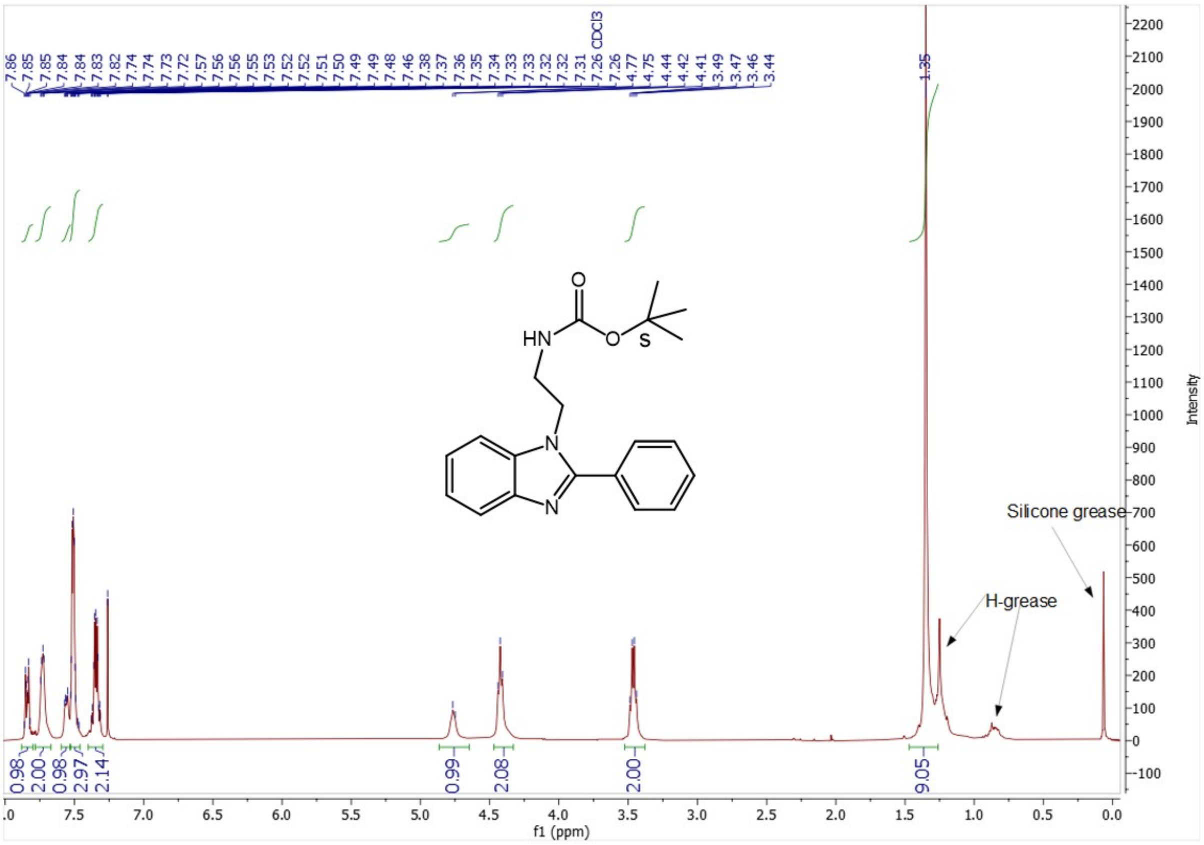

Figure S2: ^{1}H NMR spectrum of **6**

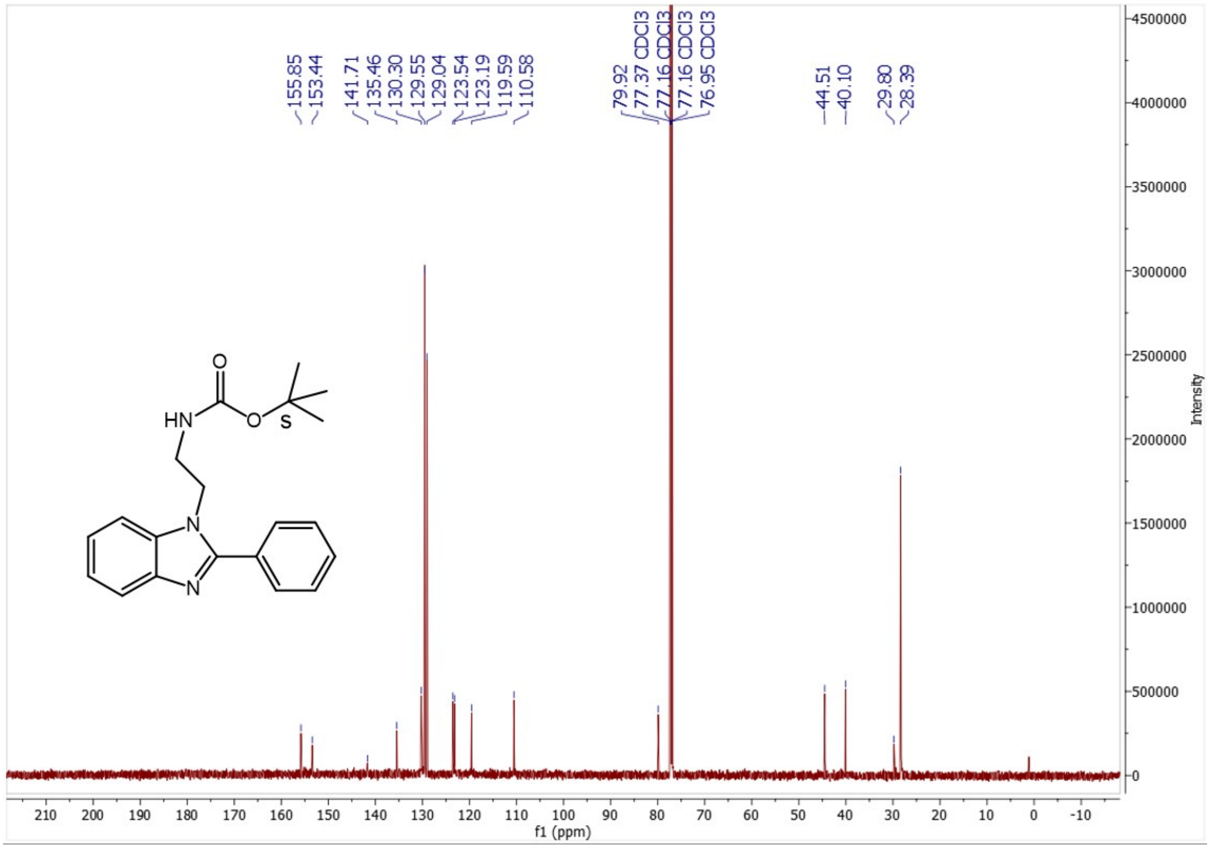

Figure S3: ^{13}NMR spectrum of **6**

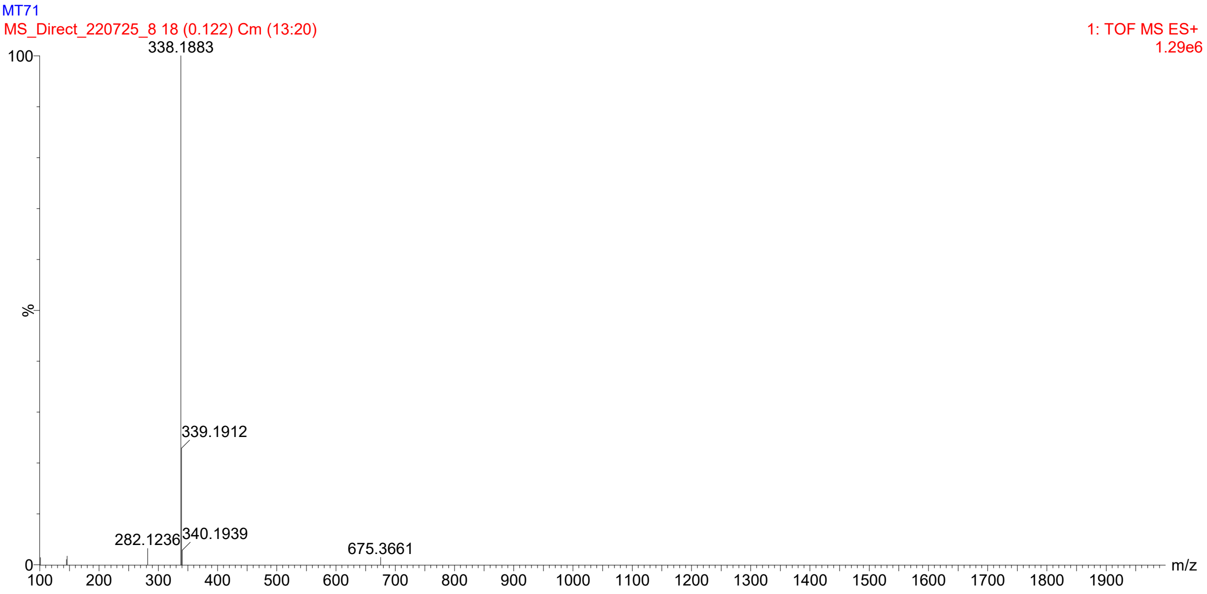

Figure S4: Experimental positive ionisation mode mass spectrum of **6**

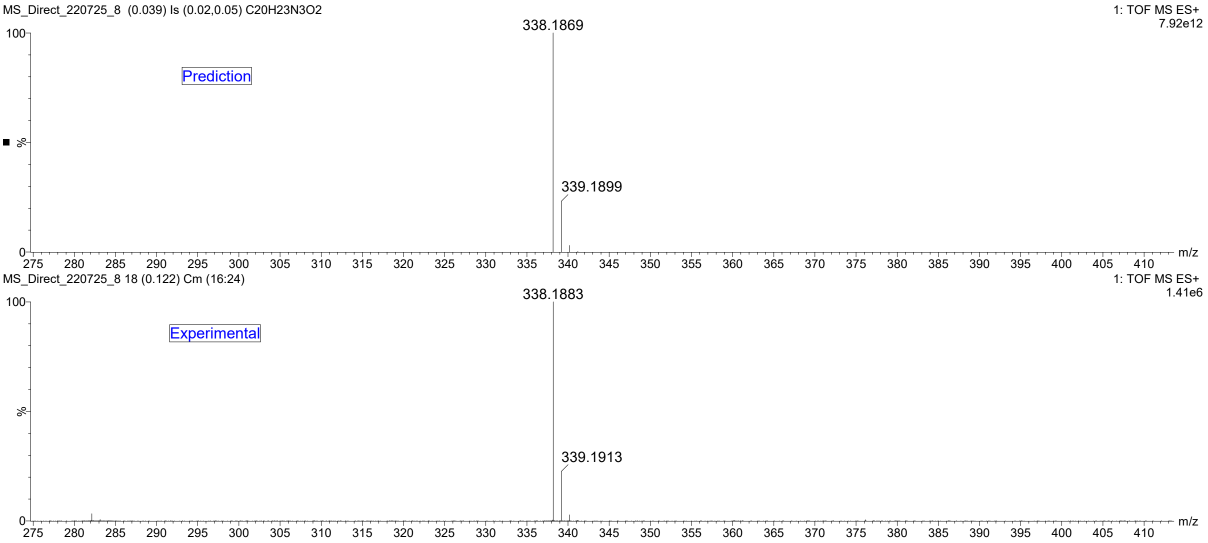

Figure S5: Predicted and experimental mass spectrum signal of **6**

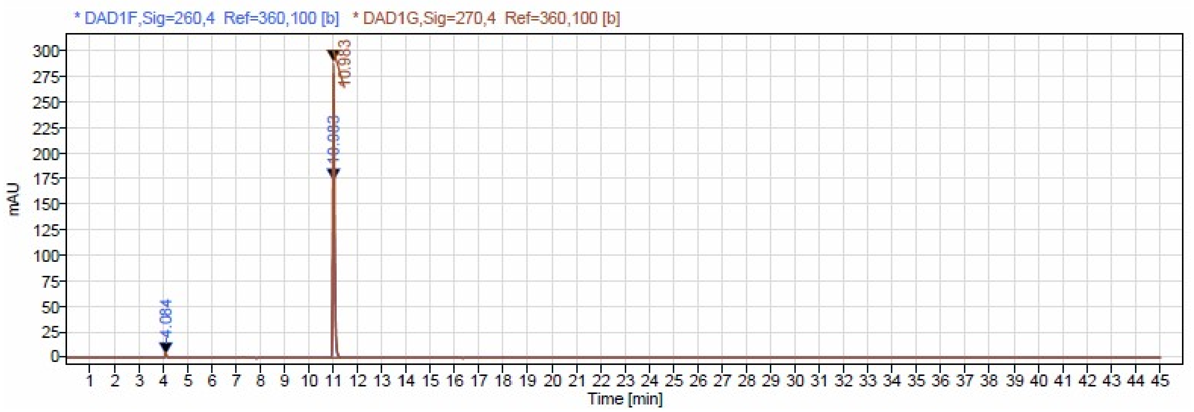

Signal: * DAD1F,Sig=260,4 Ref=360,100 [b]

RT [min]	Type	Width [min]	Area	Height	Area%	Name
4.084	MM m	0.12	9.35	2.65	0.98	
10.983	BB	0.47	945.56	173.46	99.02	
		**Sum**	**954.91**			

Figure S6: HPLC trace of **6**

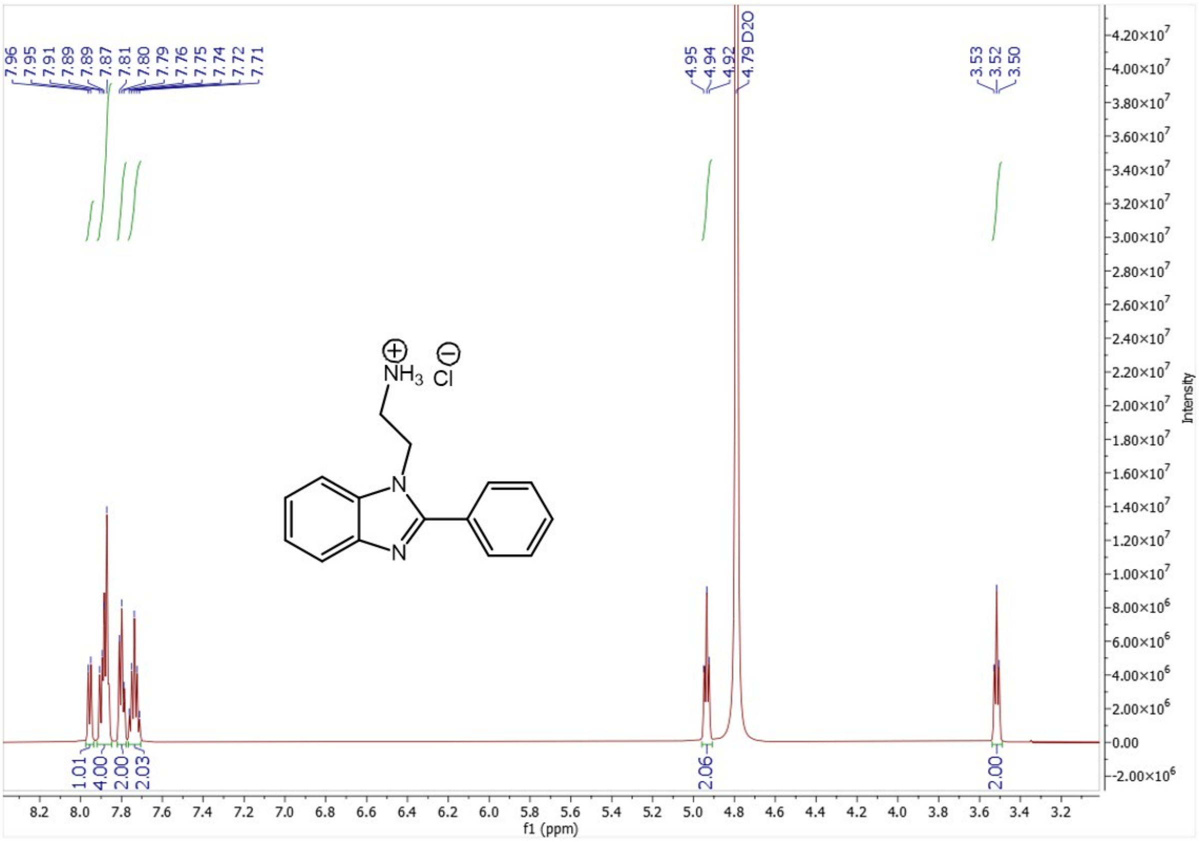

Figure S7: ^{1}H NMR spectrum of **11**

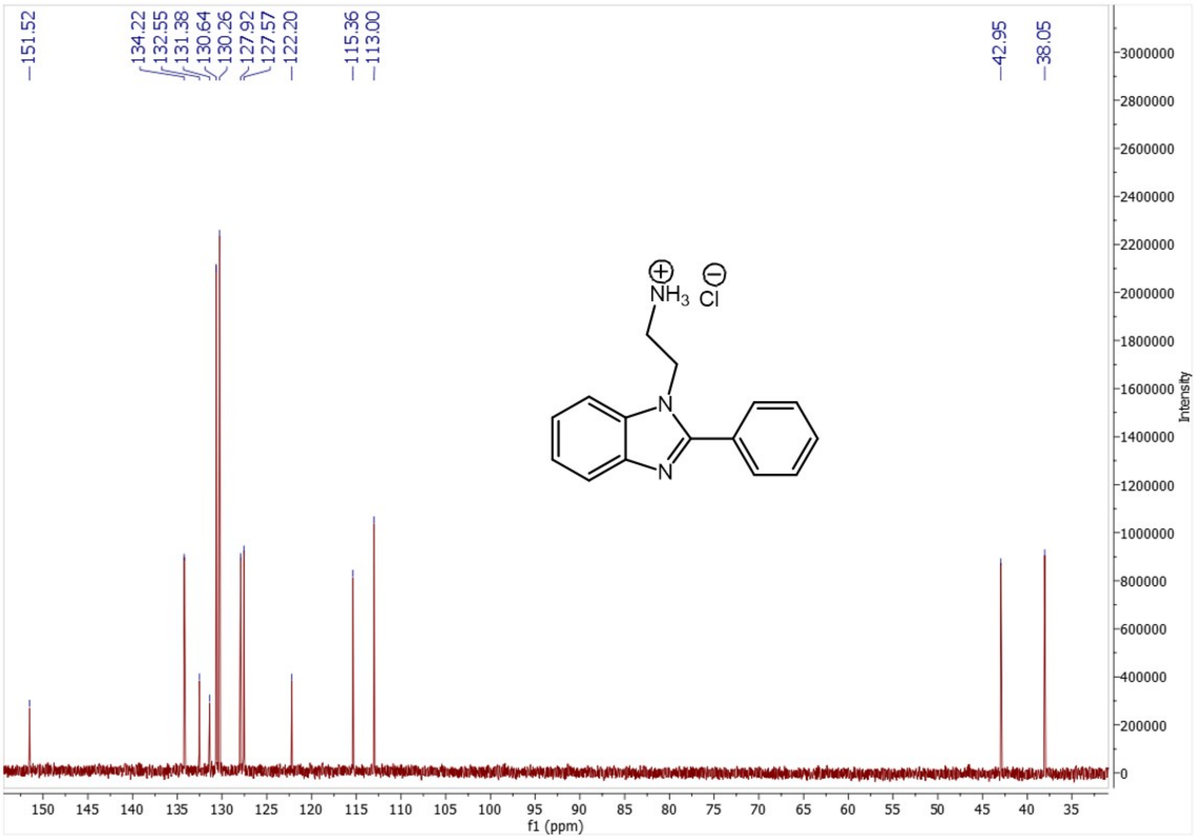

Figure S8: ^{13}NMR spectrum of **11**

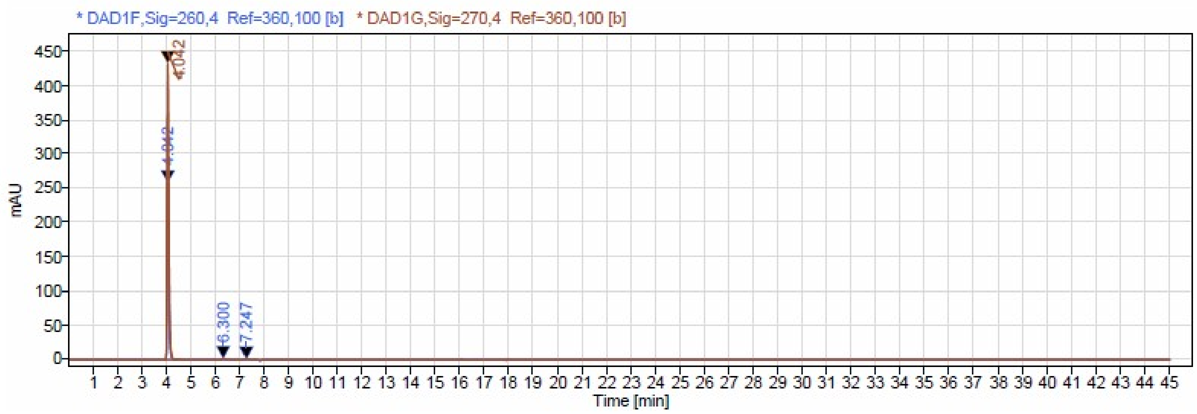

Signal:	* DAD1F,Sig=260,4 Ref=360,100 [b]					
RT [min]	Type	Width [min]	Area	Height	Area%	Name
4.042	BB	0.55	1246.28	258.71	99.41	
6.300	MM m	0.14	4.77	1.20	0.38	
7.247	MM m	0.16	2.67	0.58	0.21	
		Sum	1253.72			

Figure S9: HPLC trace of **11**

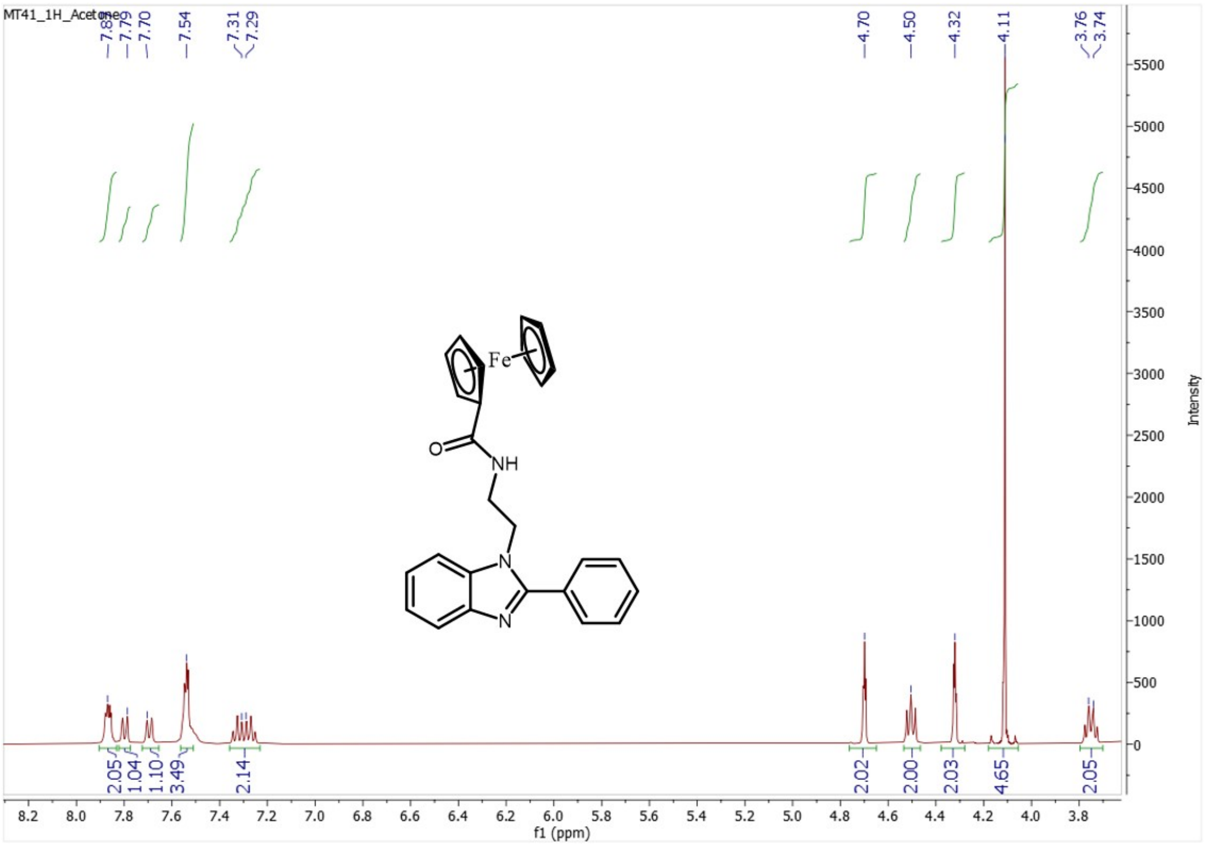

Figure S10: ^{1}H NMR spectrum of **14**

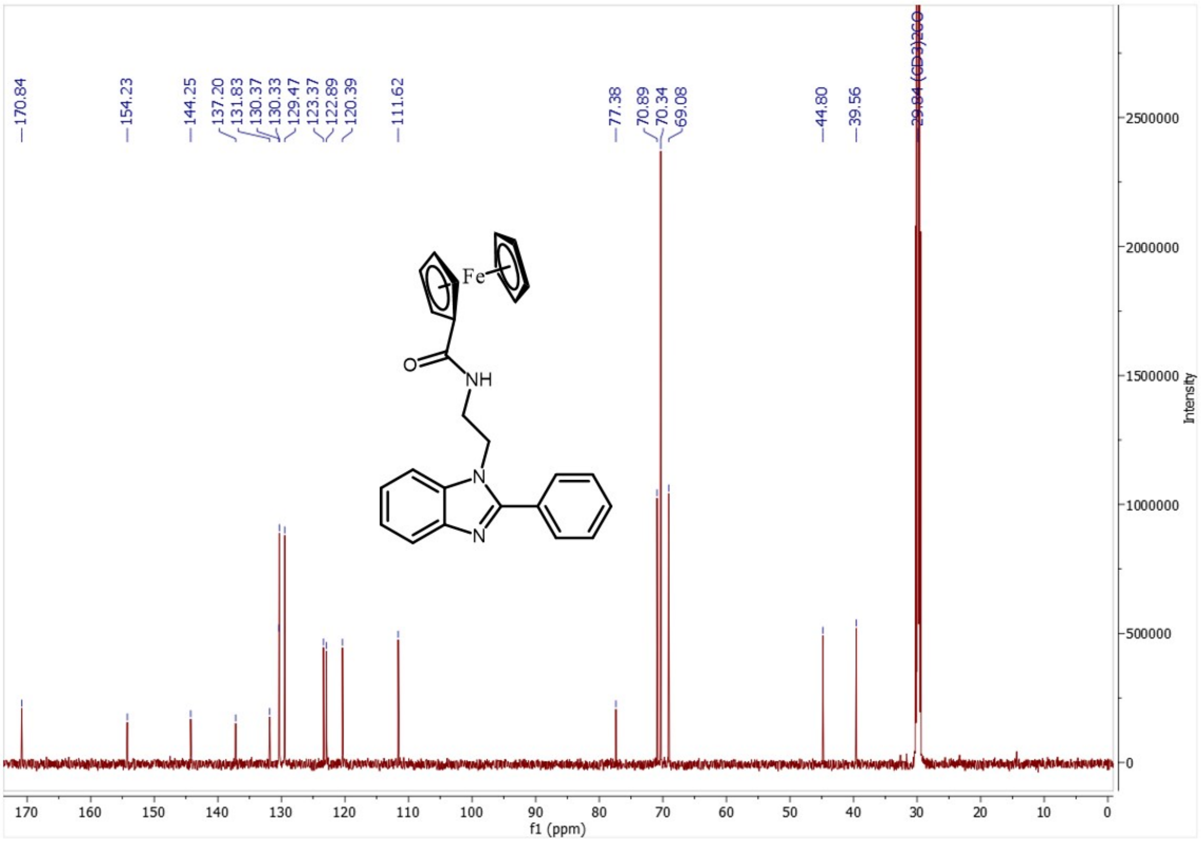

Figure S11: ^{13}C NMR spectrum of **14**

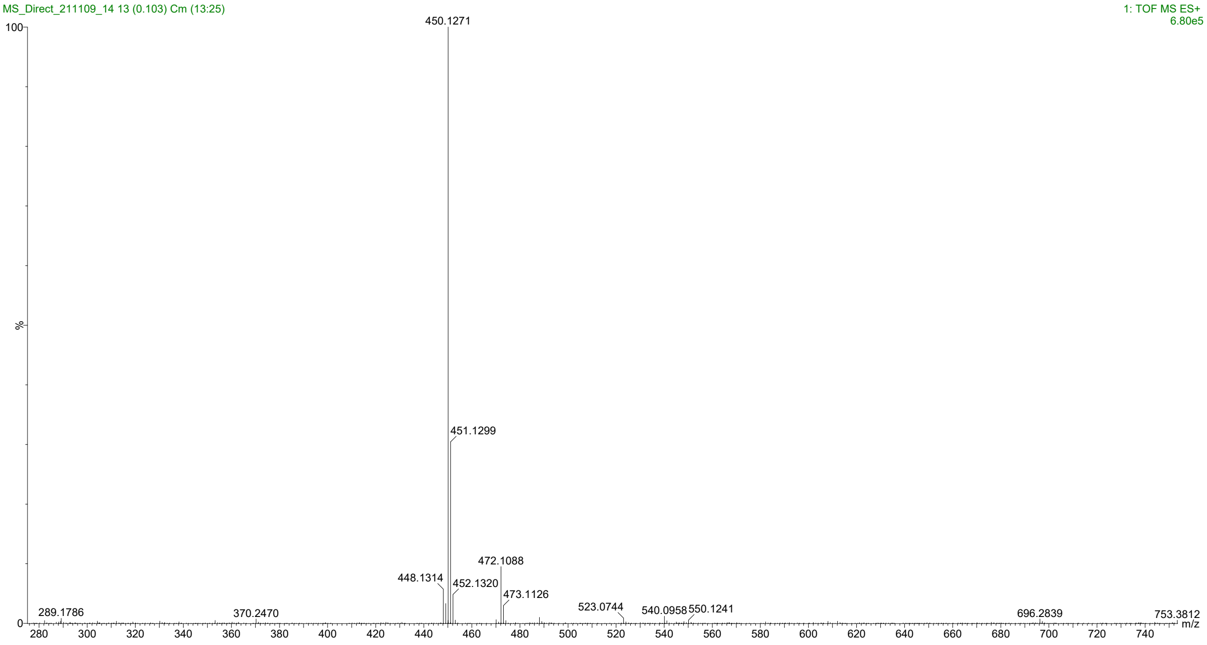

Figure S12: Experimental positive ionisation mode mass spectrum of **14**

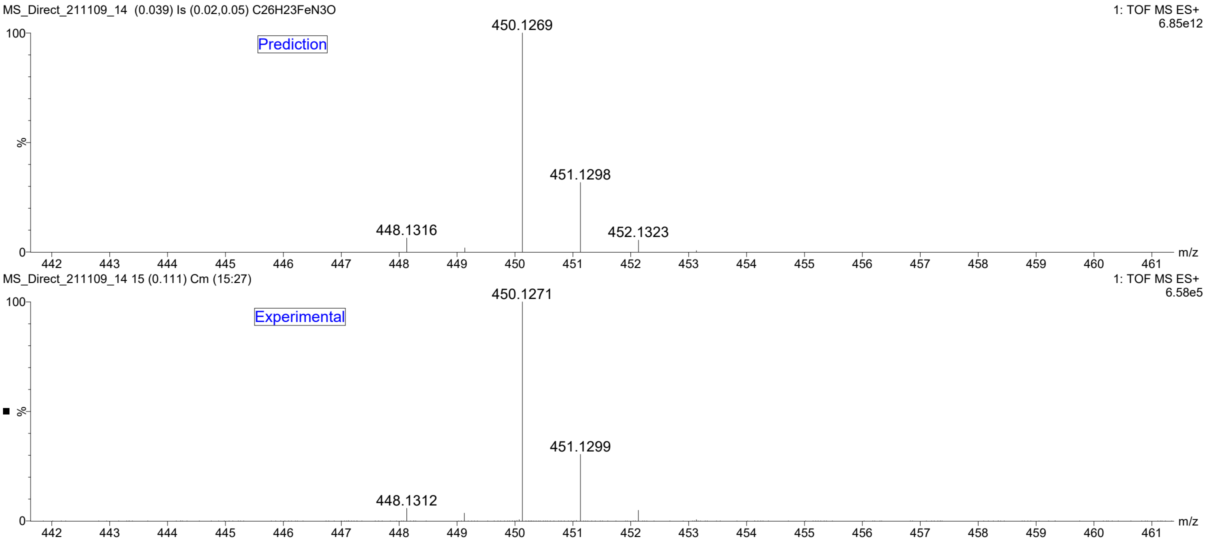

Figure S13: Predicted and experimental mass spectrum signal of **14**

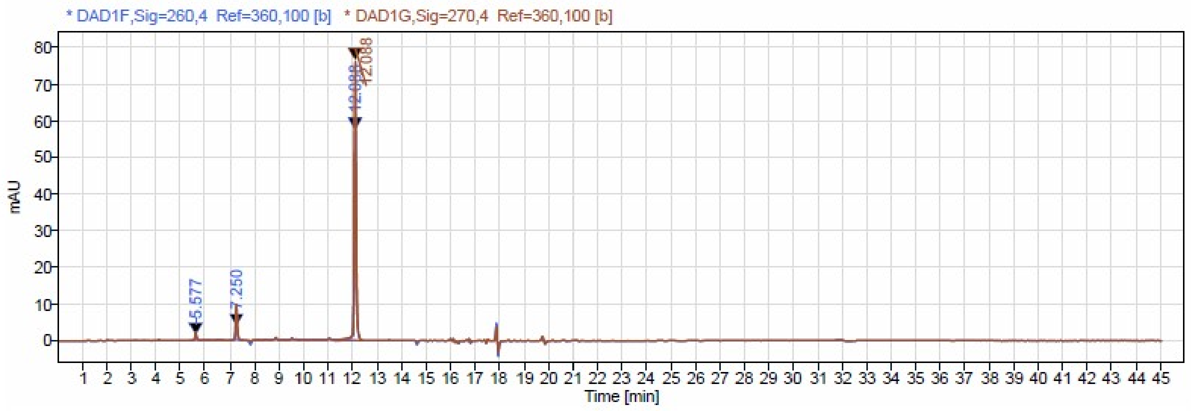

Signal: * DAD1F,Sig=260,4 Ref=360,100 [b]

RT [min]	Type	Width [min]	Area	Height	Area%	Name
5.577	MM m	0.13	5.10	1.34	1.56	
7.250	MM m	0.19	17.92	3.75	5.48	
12.088	BB	1.14	304.14	57.71	92.97	
		**Sum**	**327.16**			

Figure S14: HPLC trace of **14**

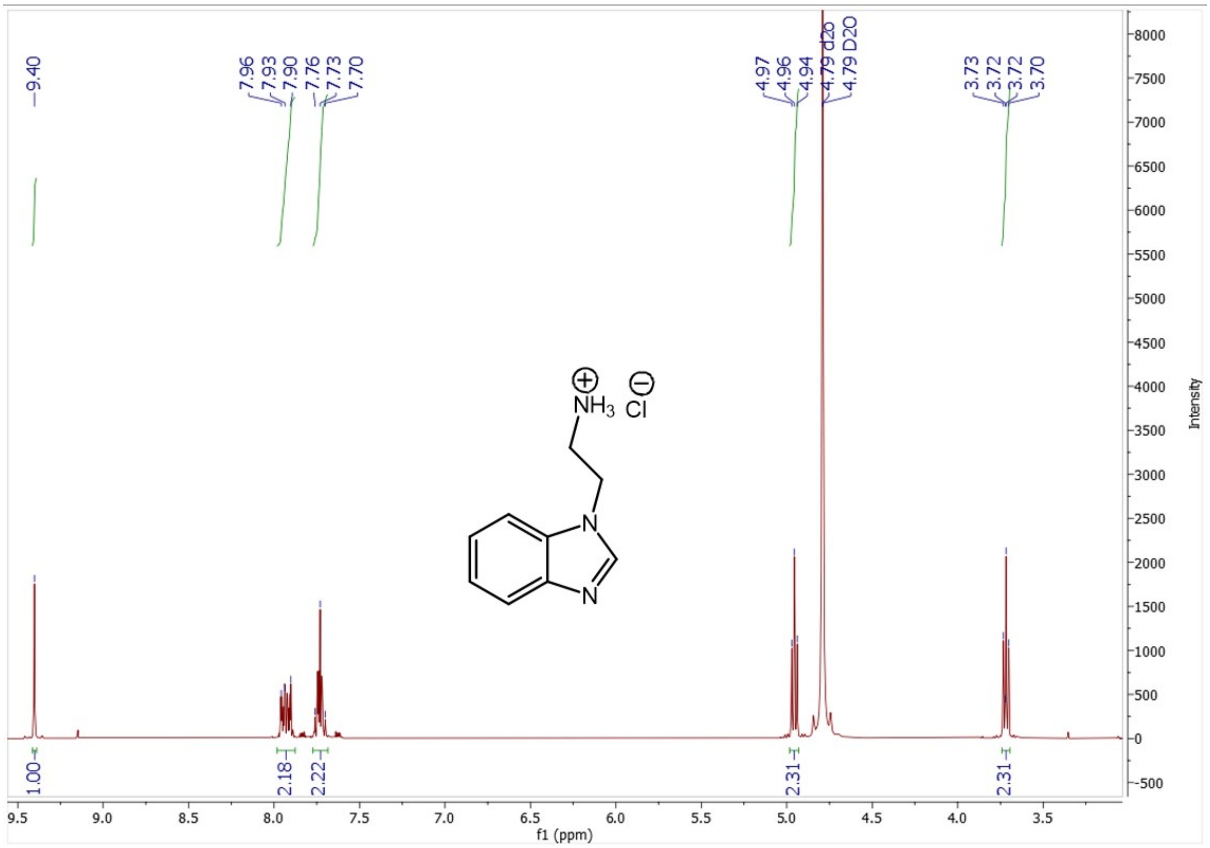

Figure S15: ^{1}H NMR spectrum of **10**

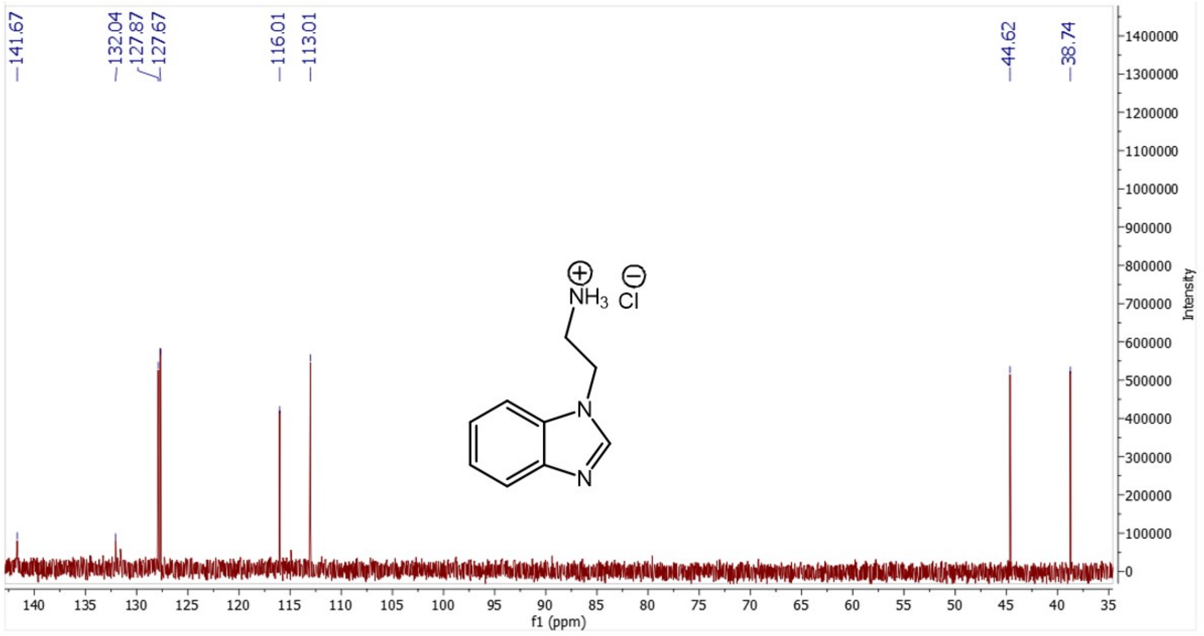

Figure S16: ^{13}C NMR spectrum of **10**

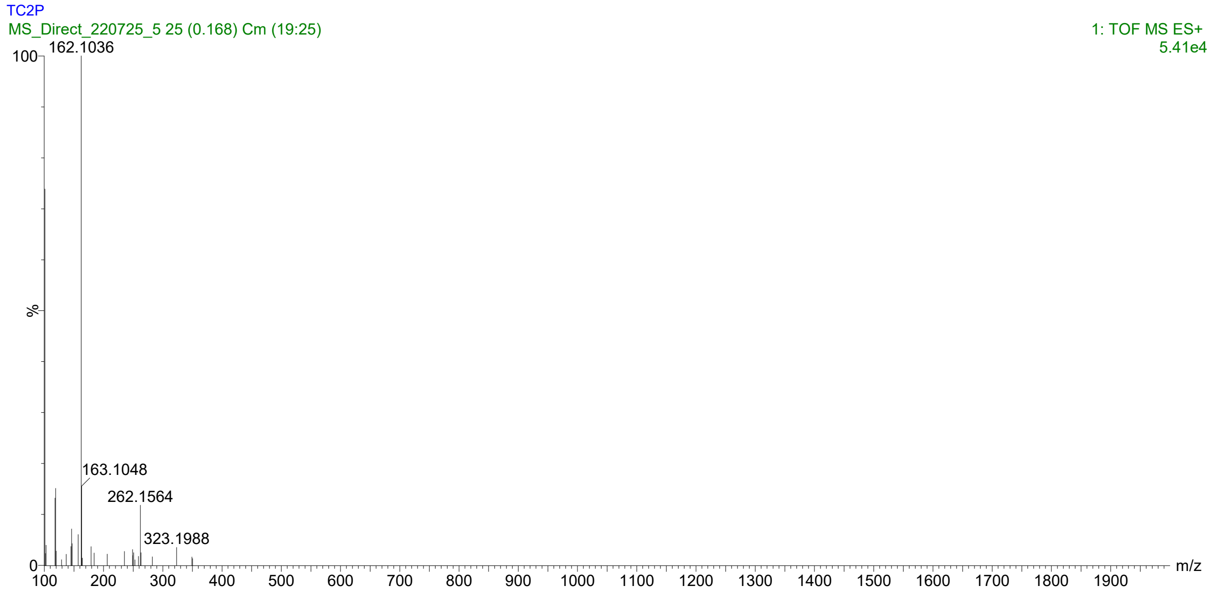

Figure S17: Experimental positive ionisation mode mass spectrum of **10**

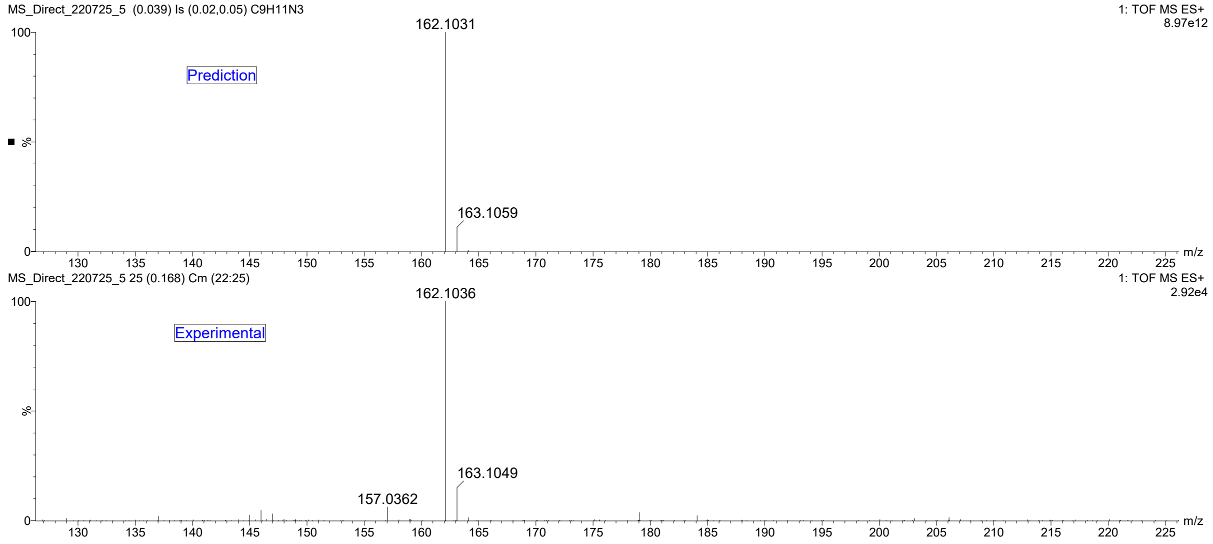

Figure S18: Predicted and experimental mass spectrum signal of **10**

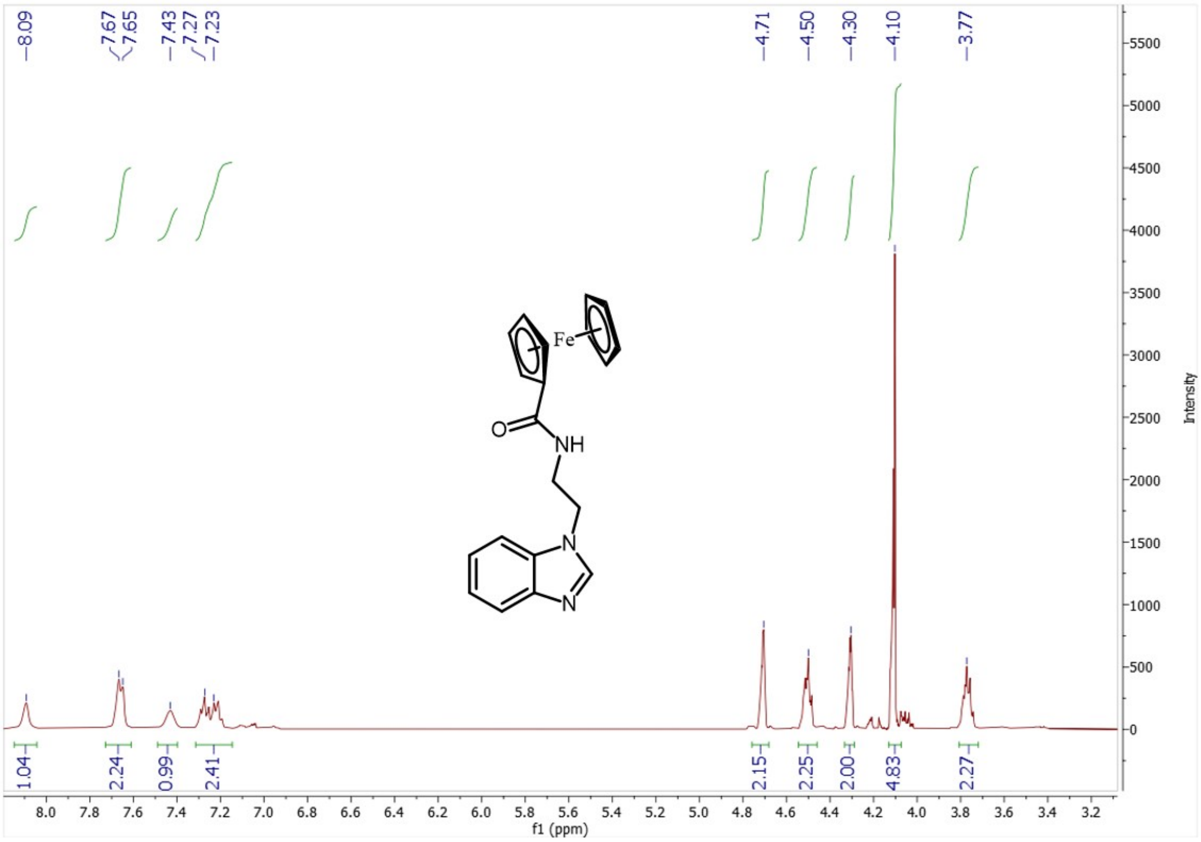

Figure S19: ^{1}H NMR spectrum of **15**

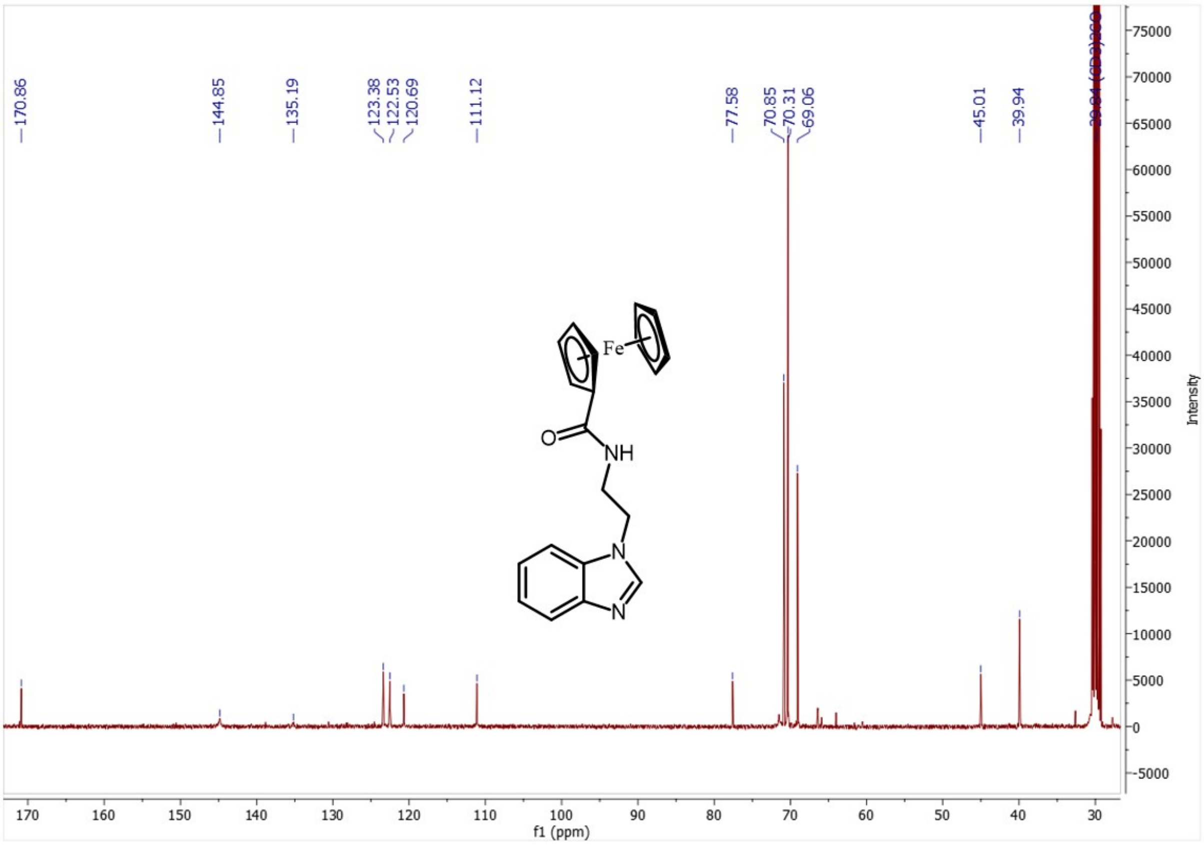

Figure S20: ^{13}C NMR spectrum of **15**

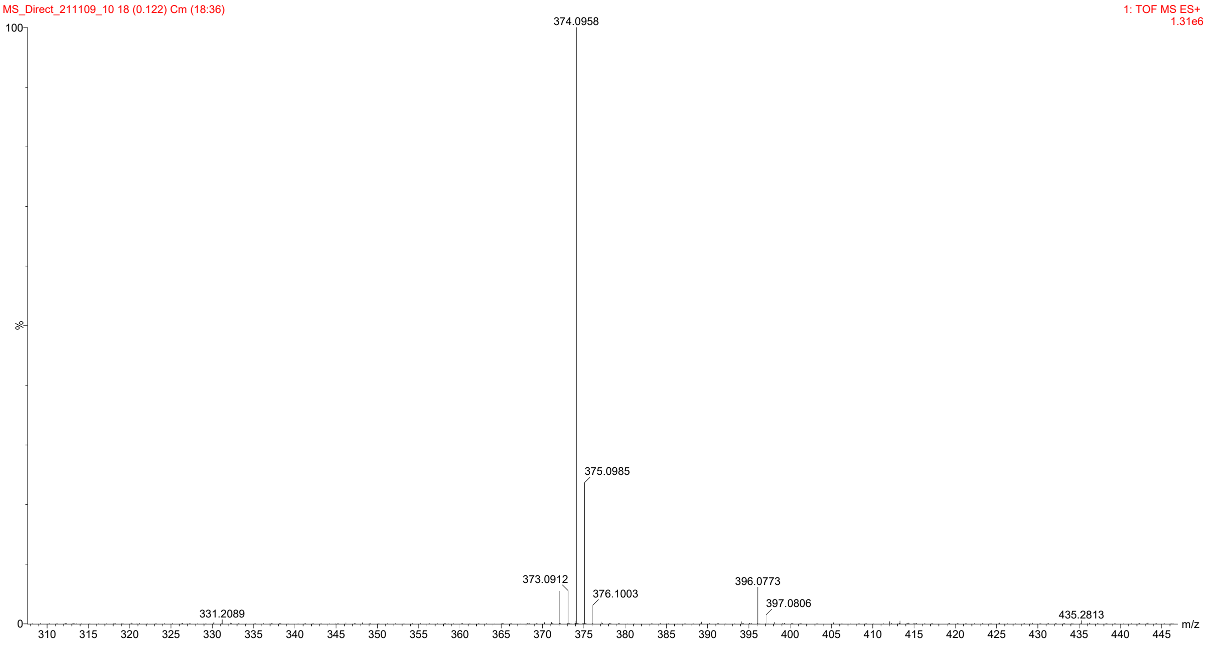

Figure S21: Experimental positive ionisation mode mass spectrum of **15**

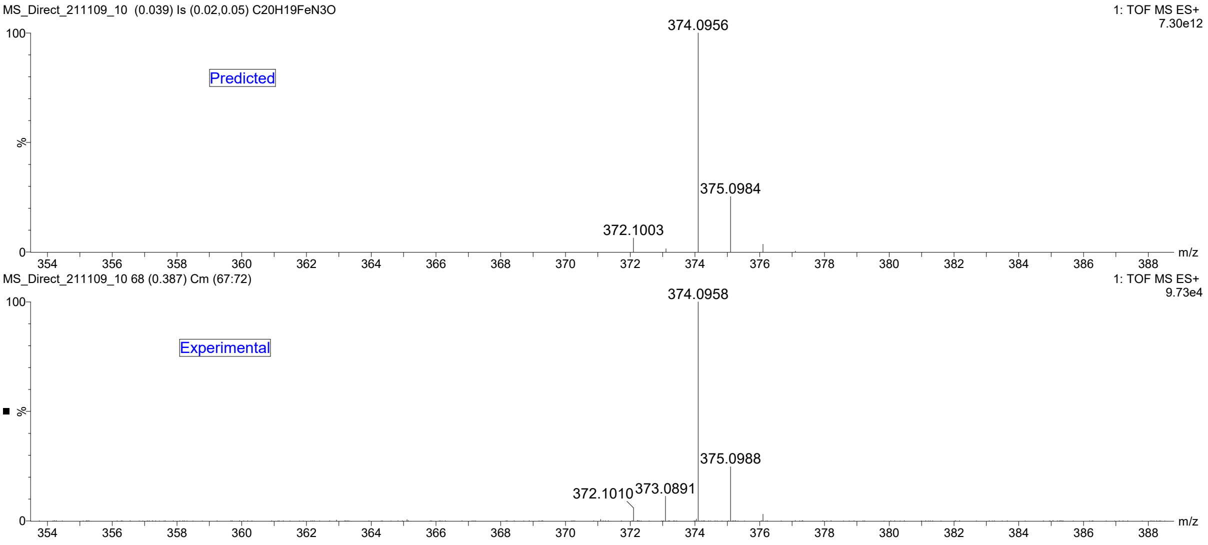

Figure S22: Predicted and experimental mass spectrum signal of **15**

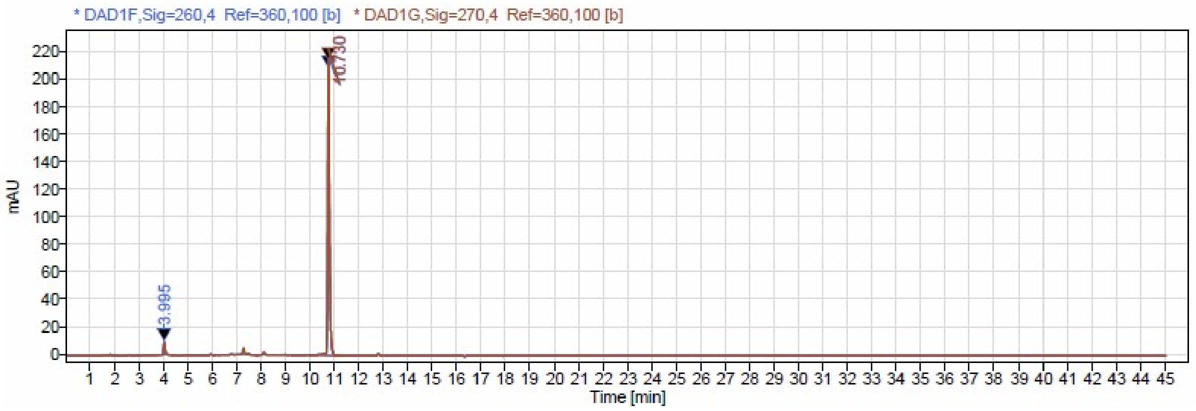

Signal: * DAD1F,Sig=260,4 Ref=360,100 [b]

RT [min]	Type	Width [min]	Area	Height	Area%	Name
3.995	MM m	0.23	41.09	9.95	3.57	
10.730	BB	1.32	1109.34	208.33	96.43	
		**Sum**	**1150.43**			

Figure S23: HPLC trace of **15**

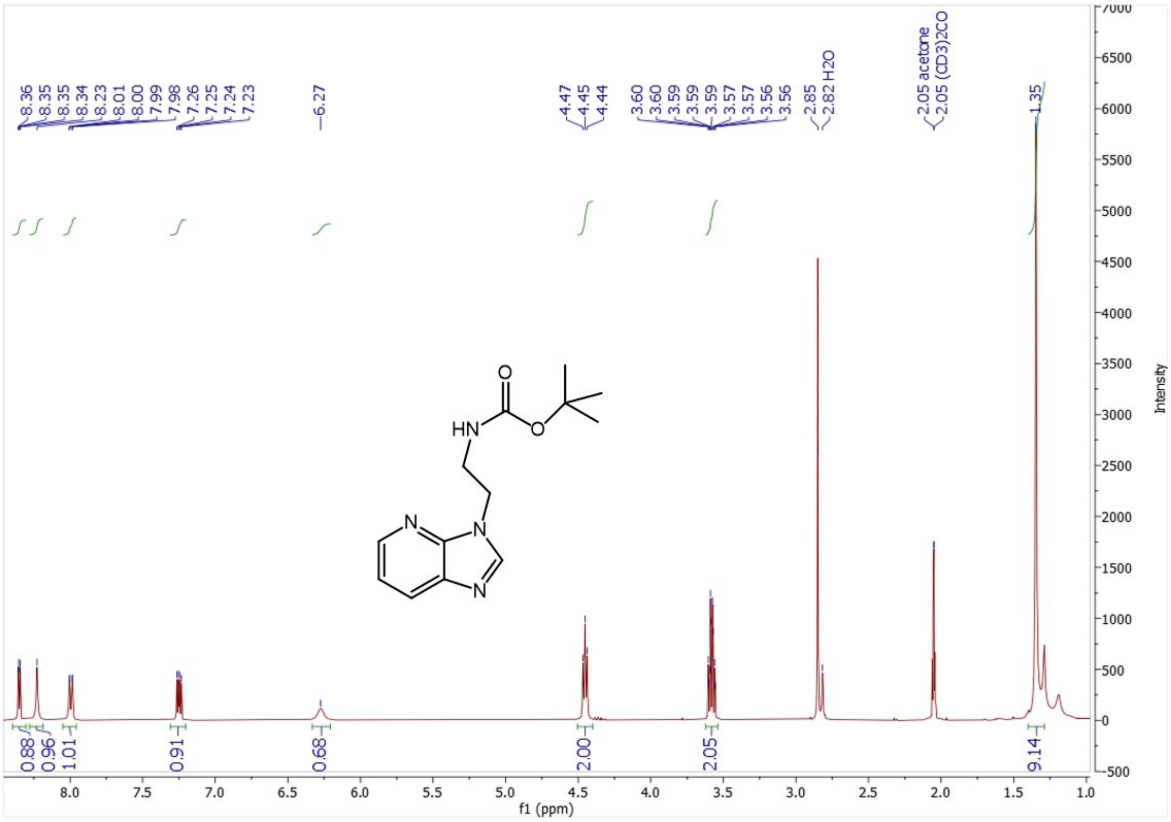

Figure S24: ^{1}H NMR spectrum of **9**

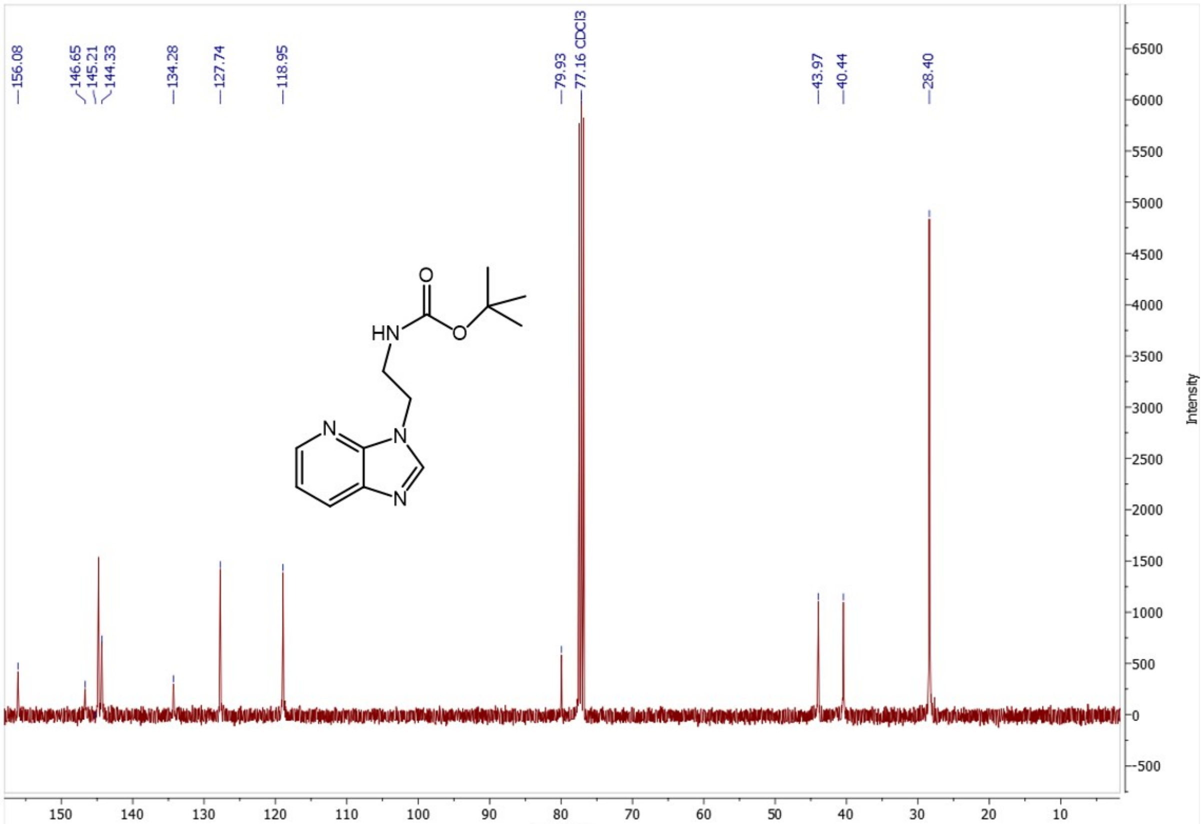

Figure S25: ^{13}C NMR spectrum of **9**

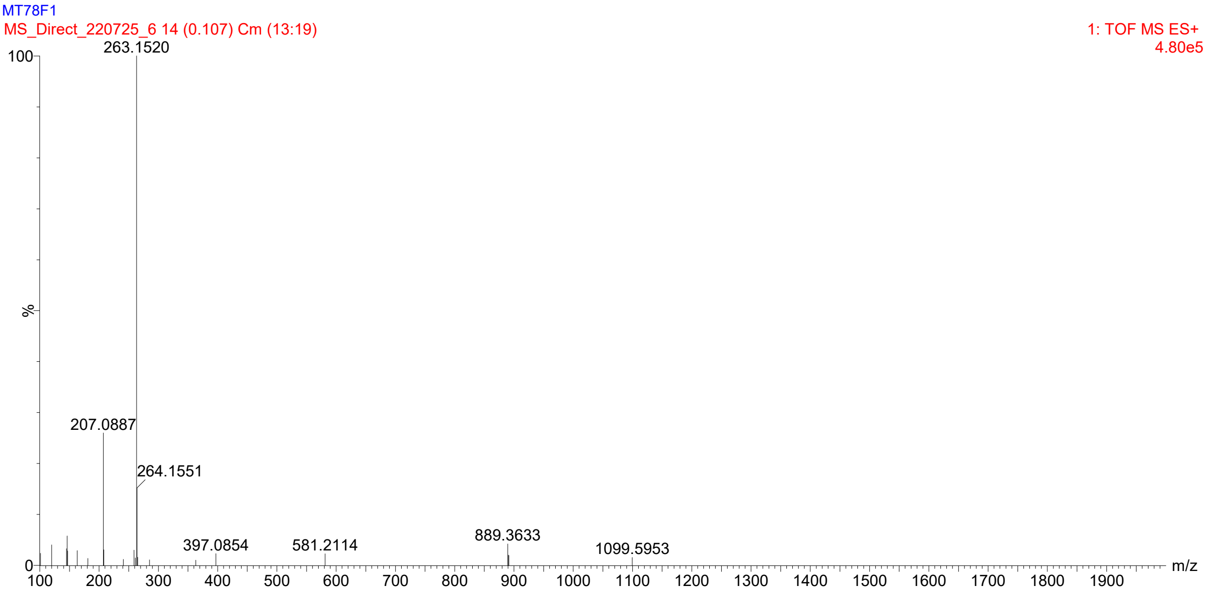

Figure S26: Experimental positive ionisation mode mass spectrum of **9**

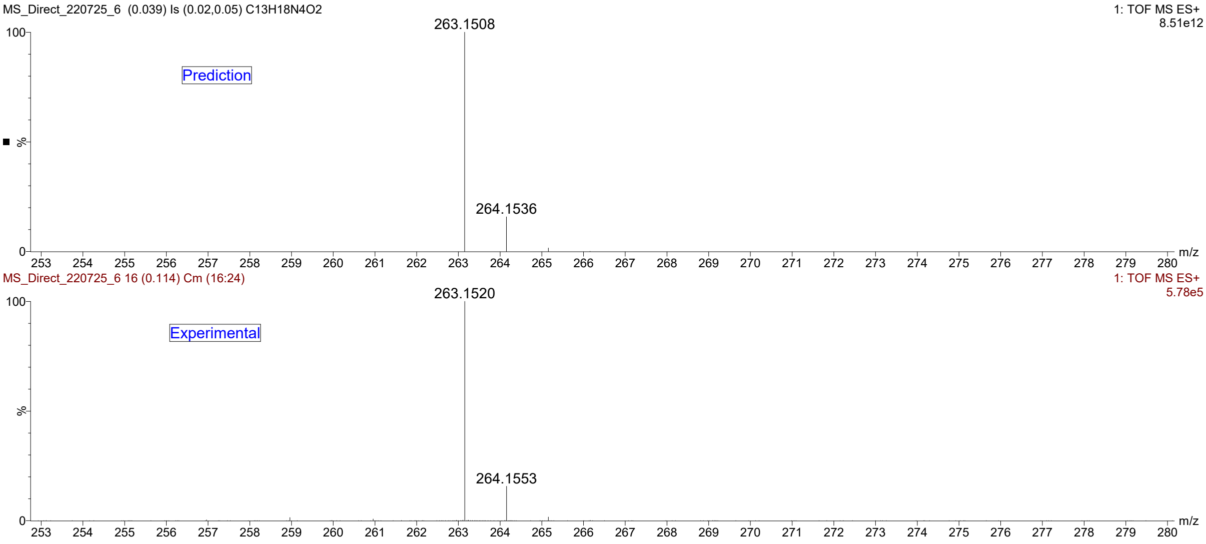

Figure 27: Predicted and experimental mass spectrum signal of **9**

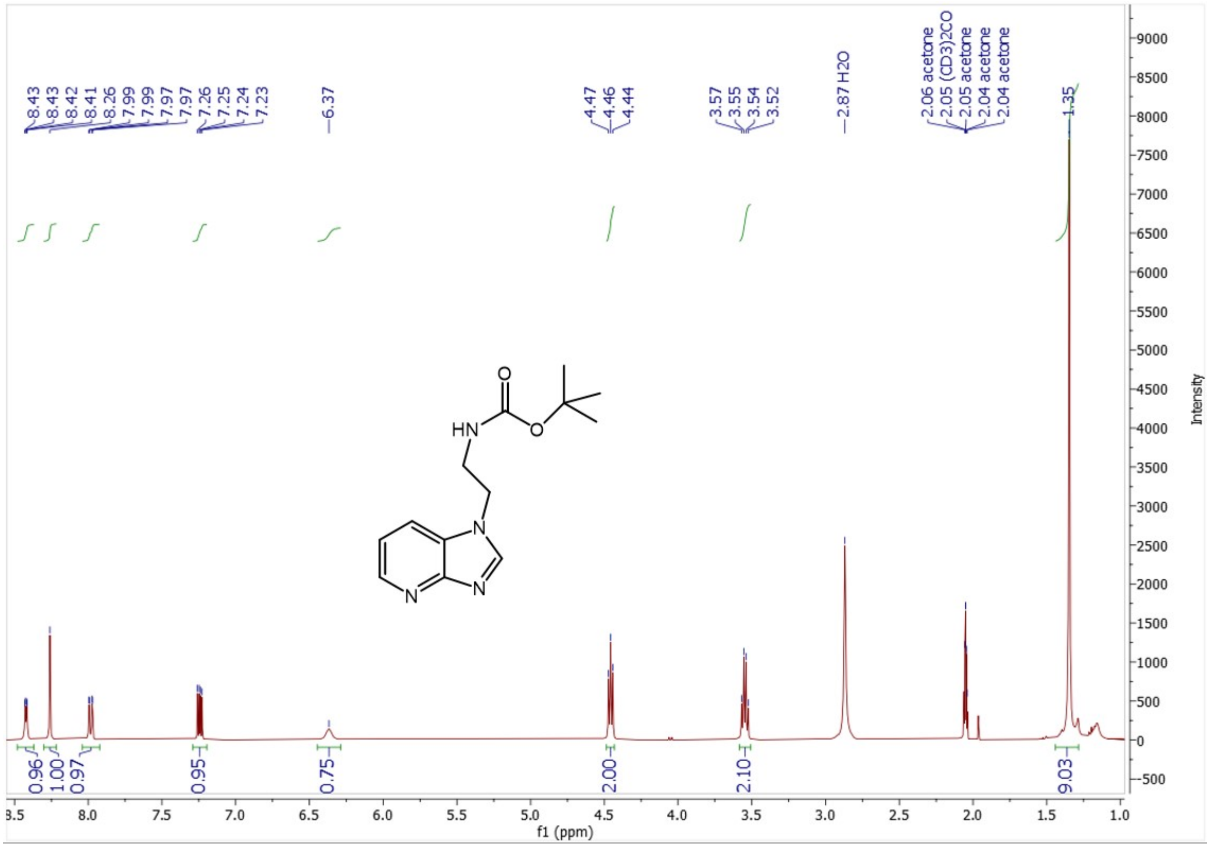

Figure S28: ^{1}H NMR spectrum of **8**

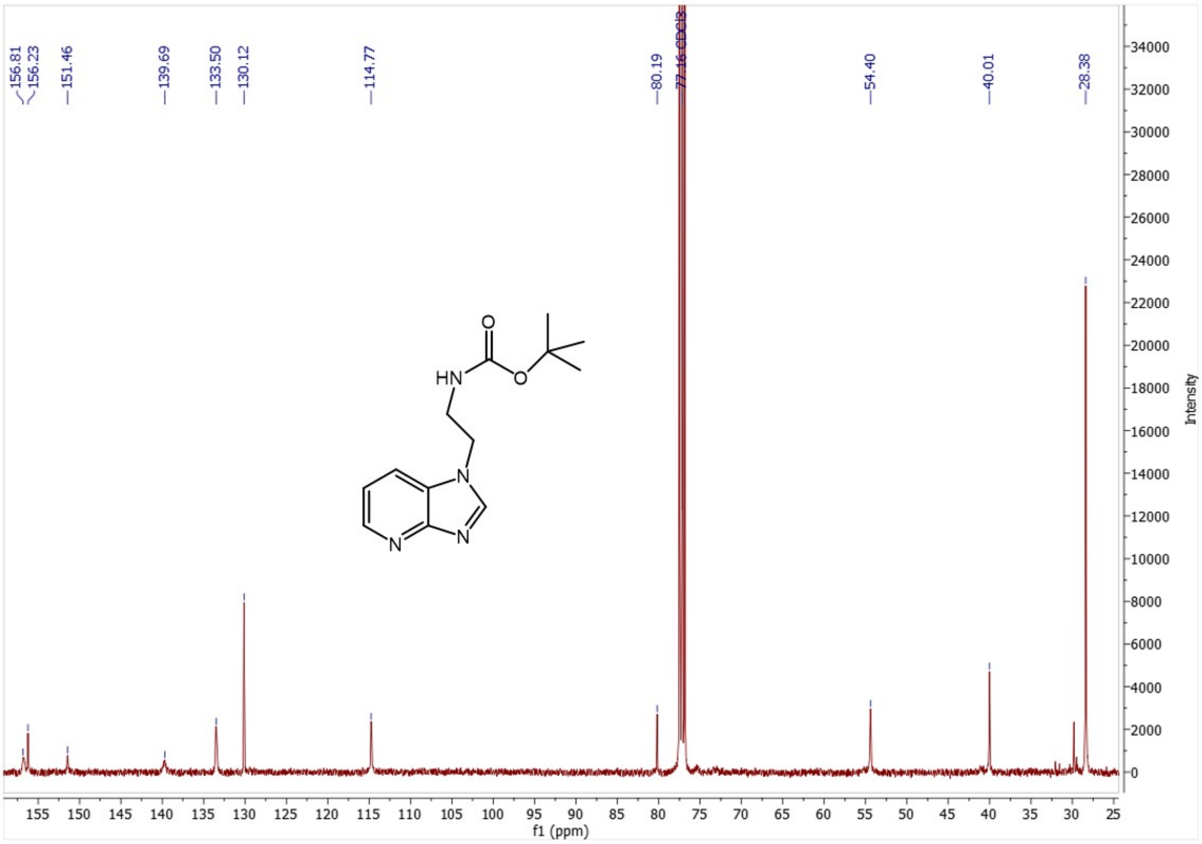

Figure S29: ^{13}C NMR spectrum of **8**

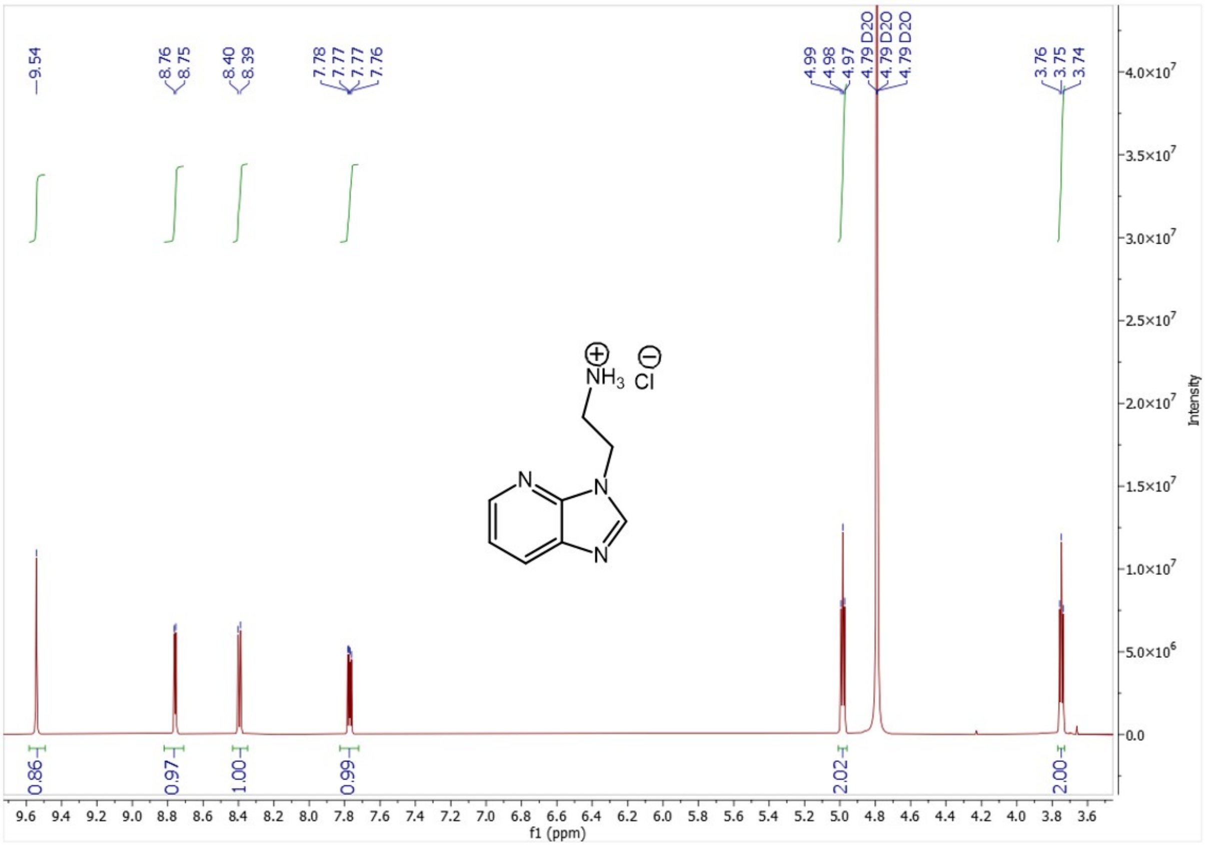

Figure S30: ^{1}H NMR spectrum of **13**

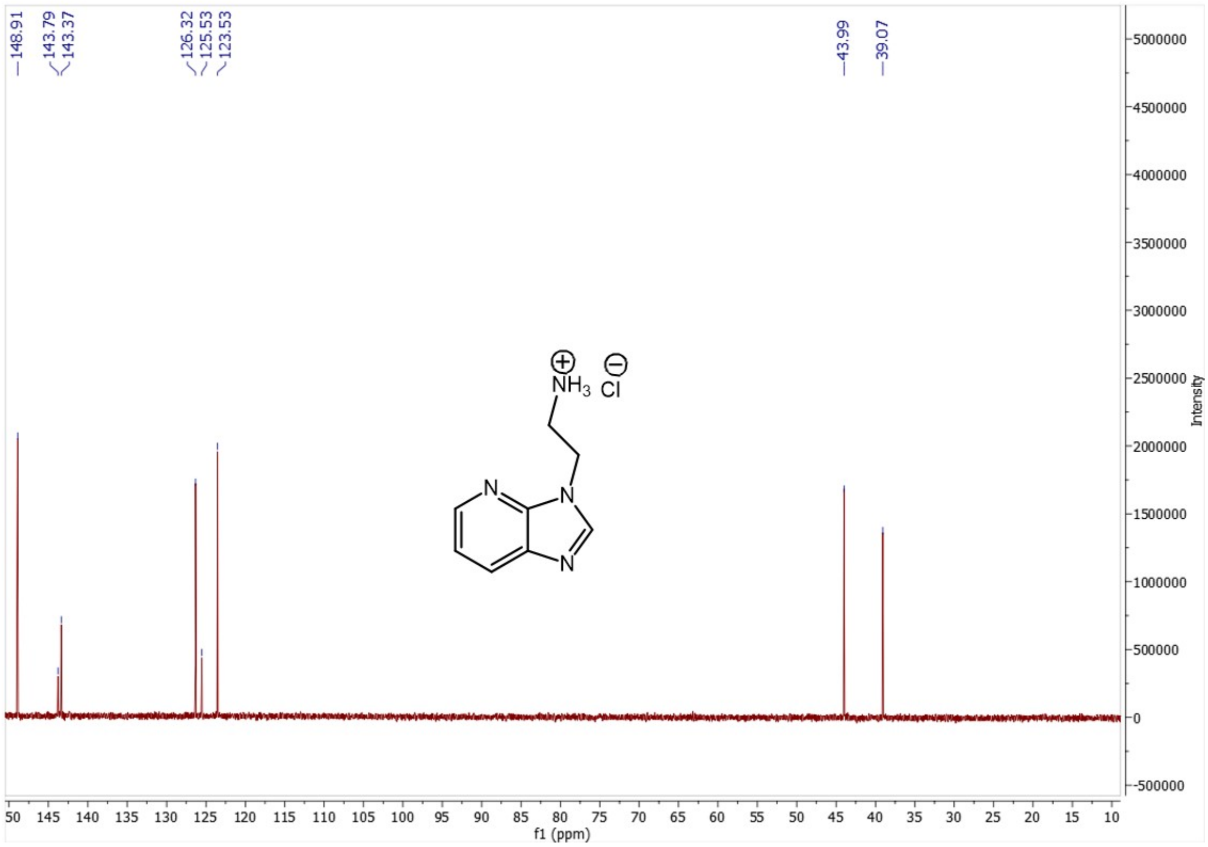

Figure S31: ^{13}C NMR spectrum of **13**

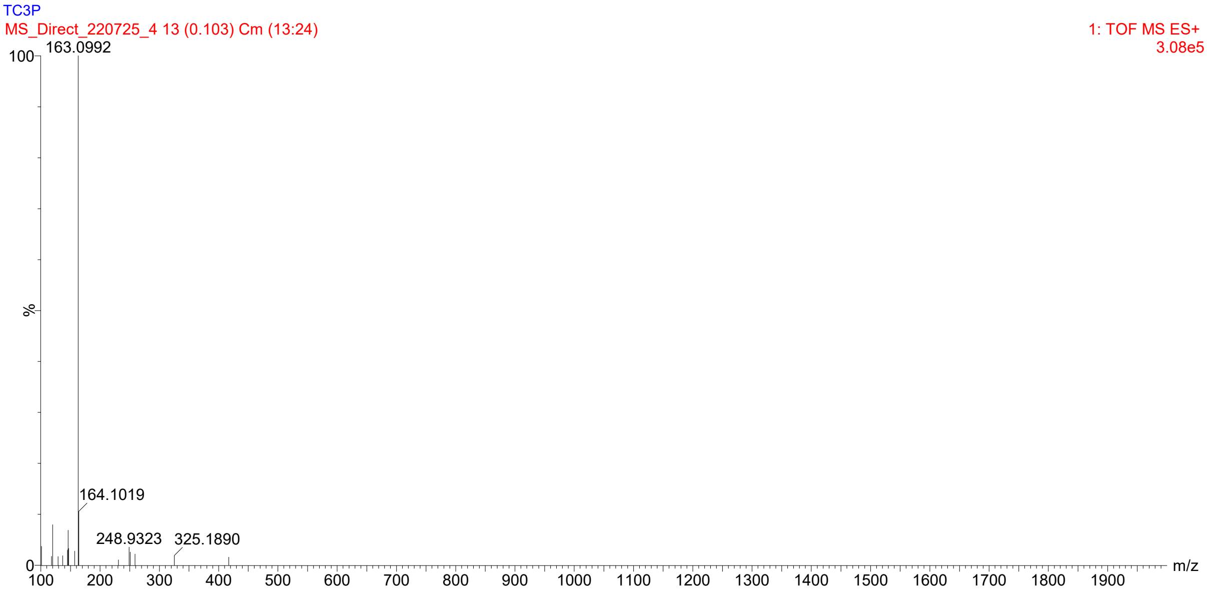

Figure S32: Experimental positive ionisation mode mass spectrum of **13**

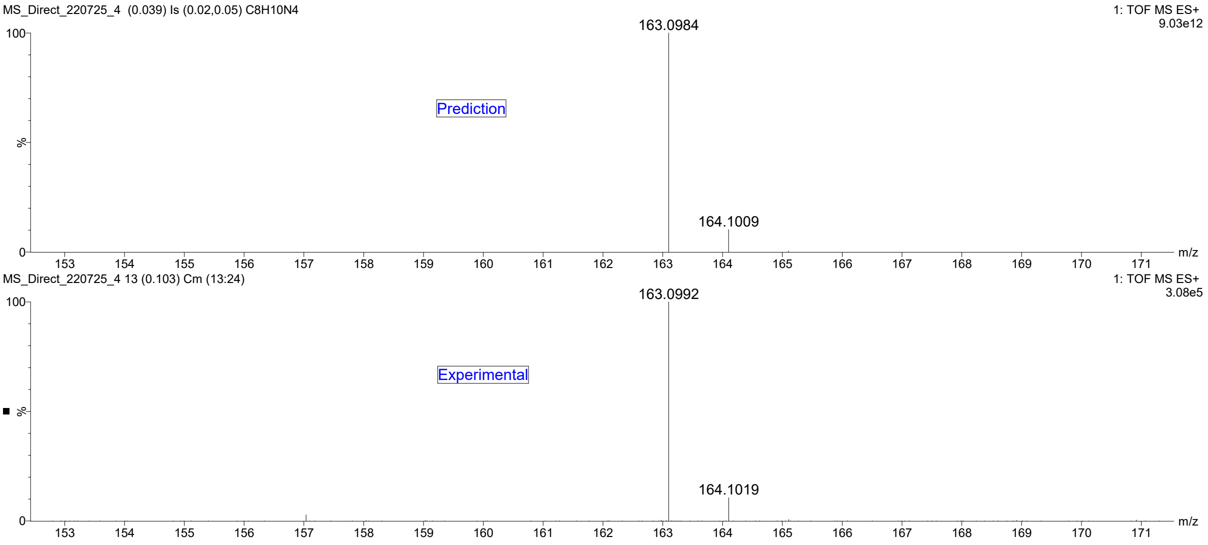

Figure S33: Predicted and experimental mass spectrum signal of **13**

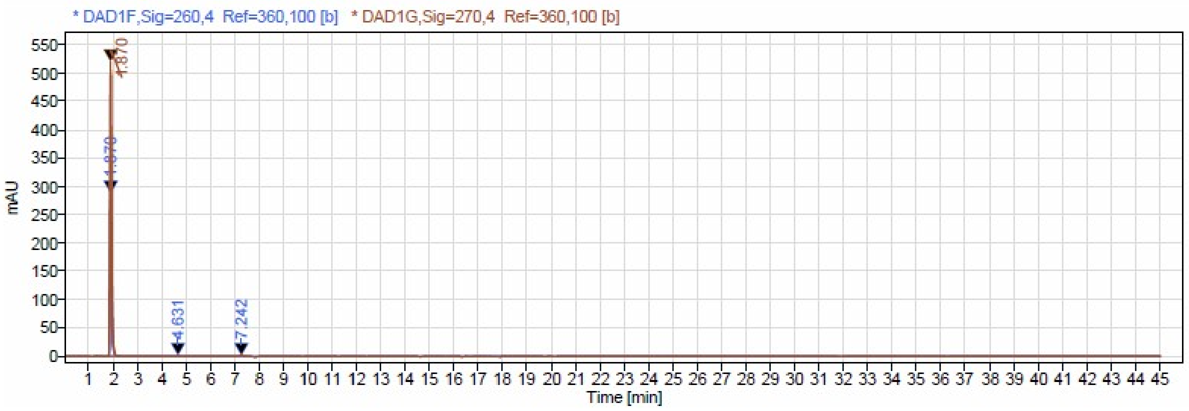

Signal: * DAD1F,Sig=260,4 Ref=360,100 [b]

RT [min]	Type	Width [min]	Area	Height	Area%	Name
1.870	VV	0.48	1196.41	288.99	98.97	
4.631	MM m	0.13	5.70	1.51	0.47	
7.242	MM m	0.17	6.72	1.44	0.56	
		Sum	1208.83			

Figure S34: HPLC trace of **13**

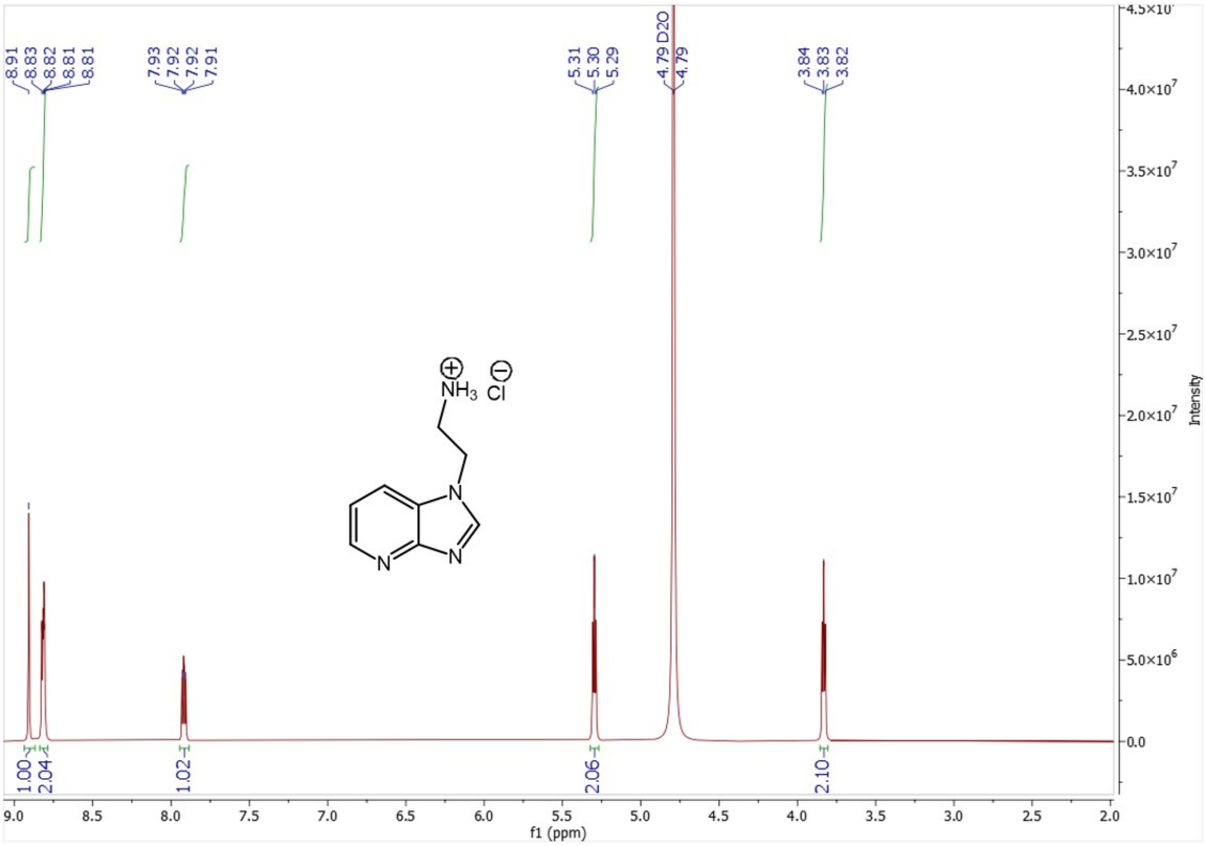

Figure S35: ^{1}H NMR spectrum of **12**

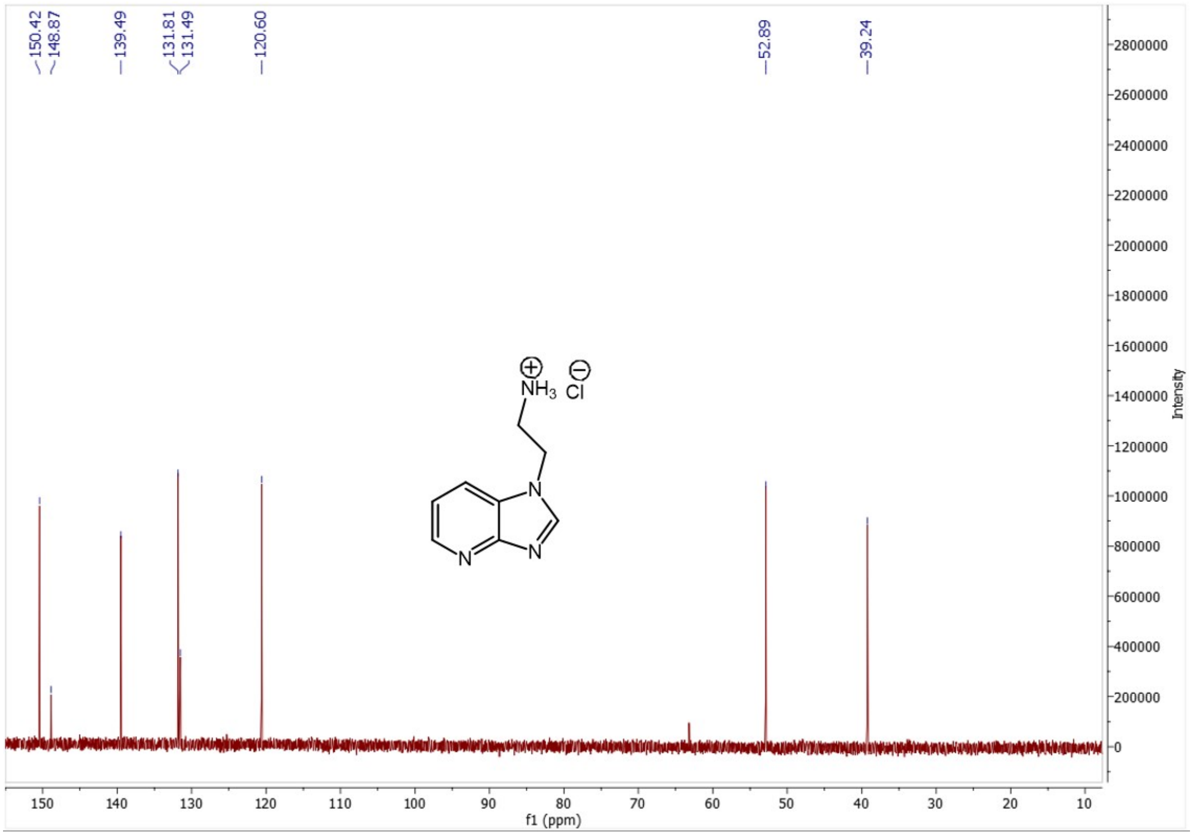

Figure S36: ^{13}C NMR spectrum of **12**

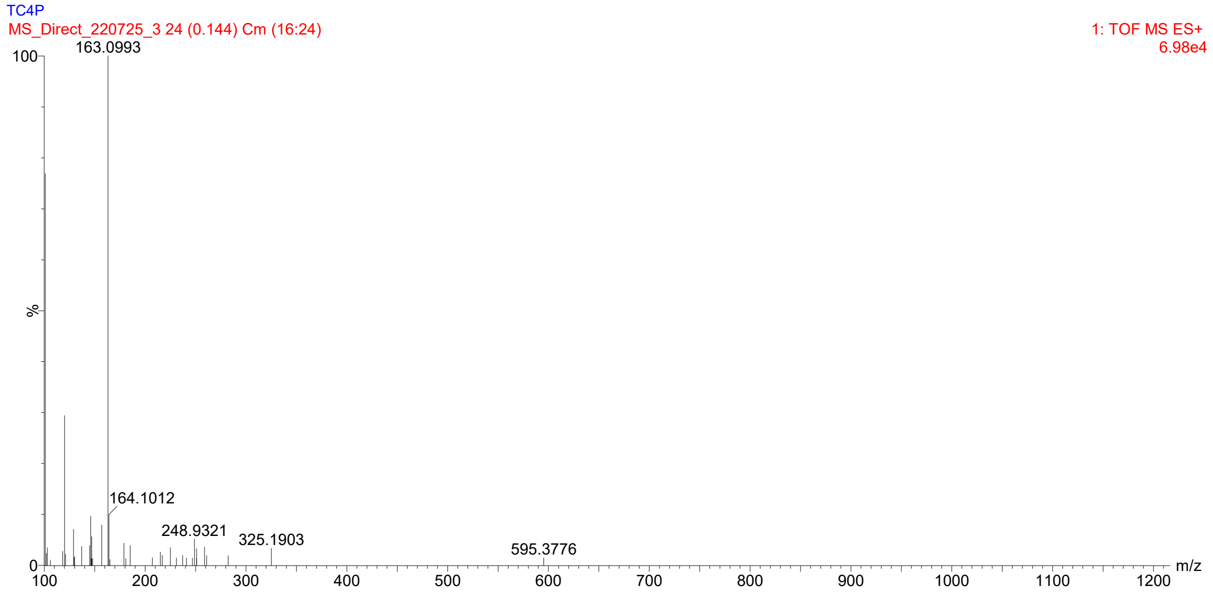

Figure S37: Experimental positive ionisation mode mass spectrum of **12**

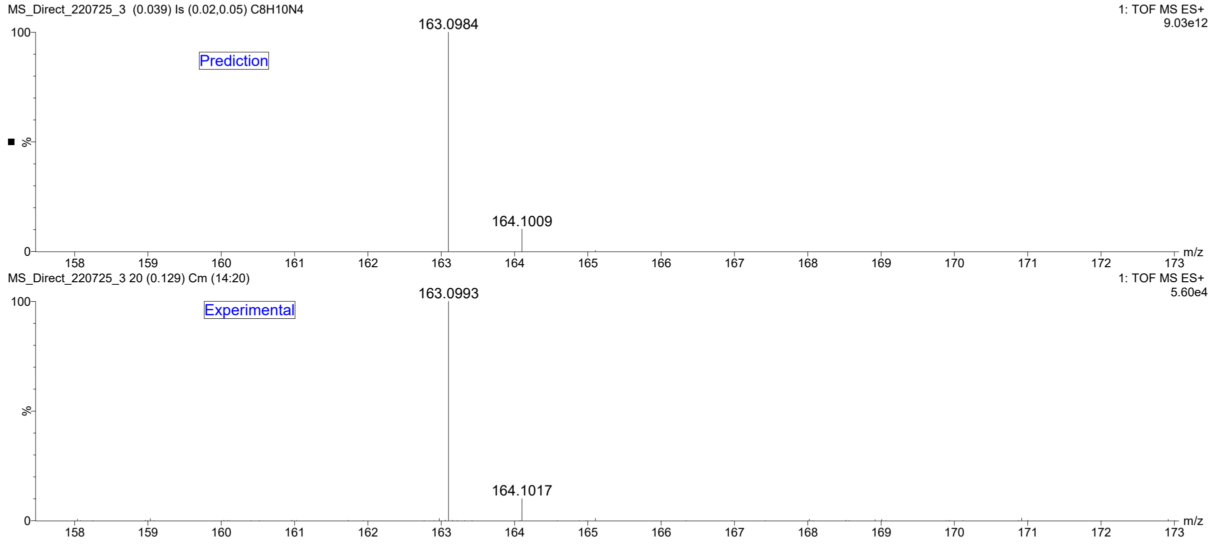

Figure 38: Predicted and experimental mass spectrum signal of **12**

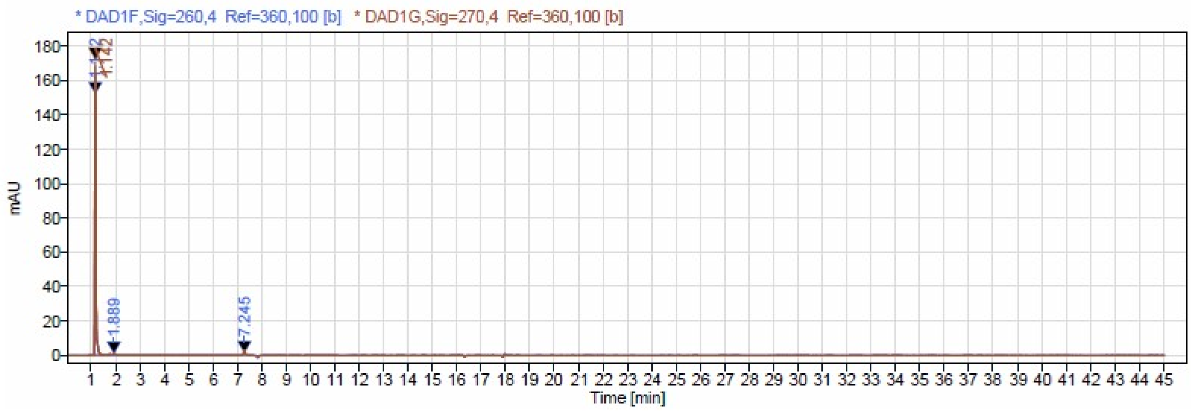

Signal: * DAD1F,Sig=260,4 Ref=360,100 [b]

RT [min]	Type	Width [min]	Area	Height	Area%	Name
1.142	BB	0.54	457.76	152.19	97.34	
1.889	MM m	0.38	7.23	0.76	1.54	
7.245	MM m	0.18	5.28	1.12	1.12	
		Sum	470.28			

Figure S39: HPLC trace of **12**

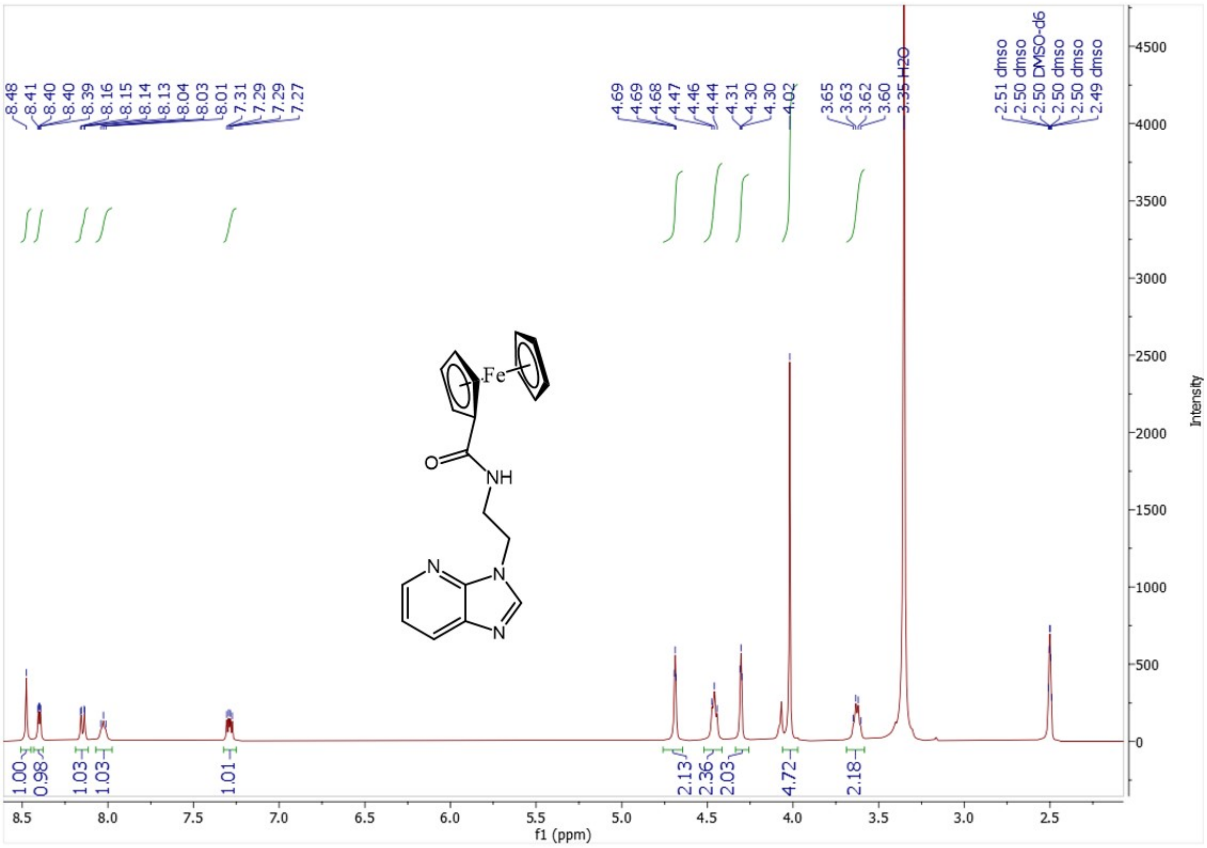

Figure S40: ^{1}H NMR spectrum of **17**

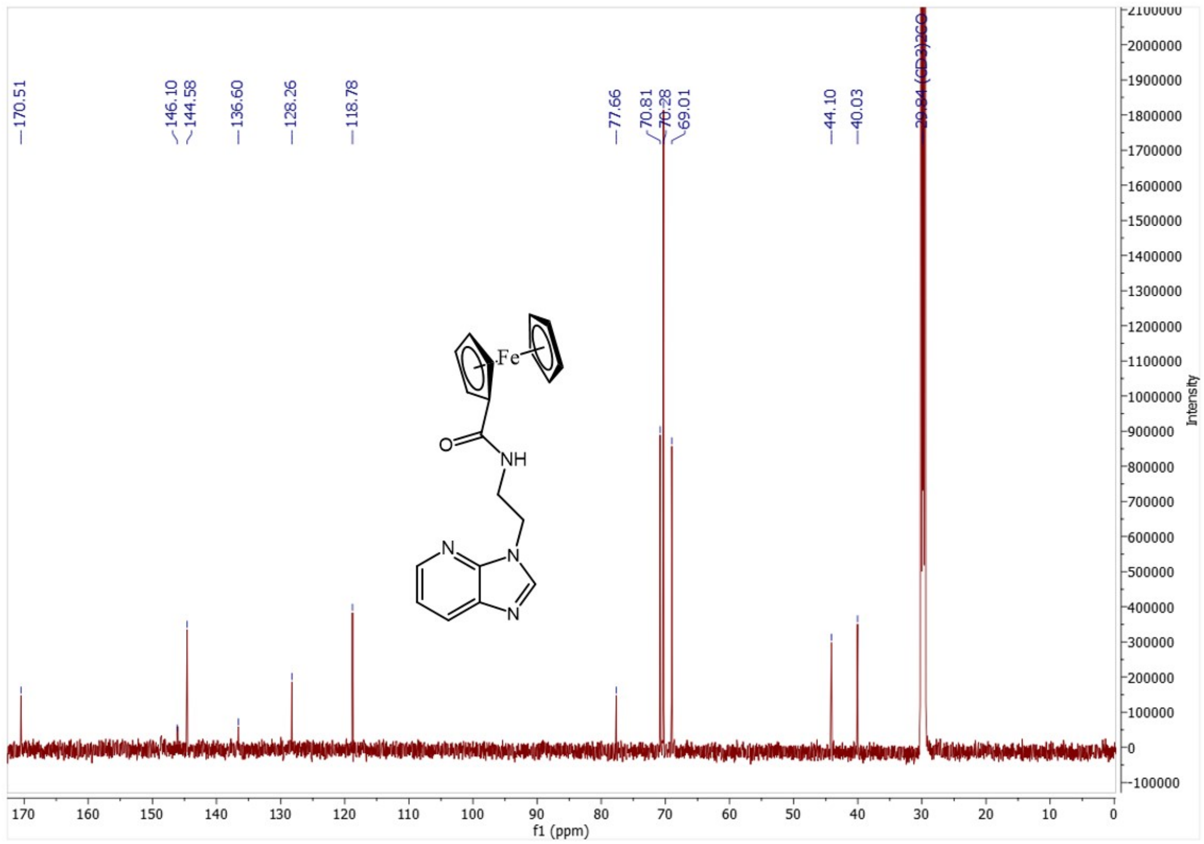

Figure S41: ^{13}C NMR spectrum of **17**

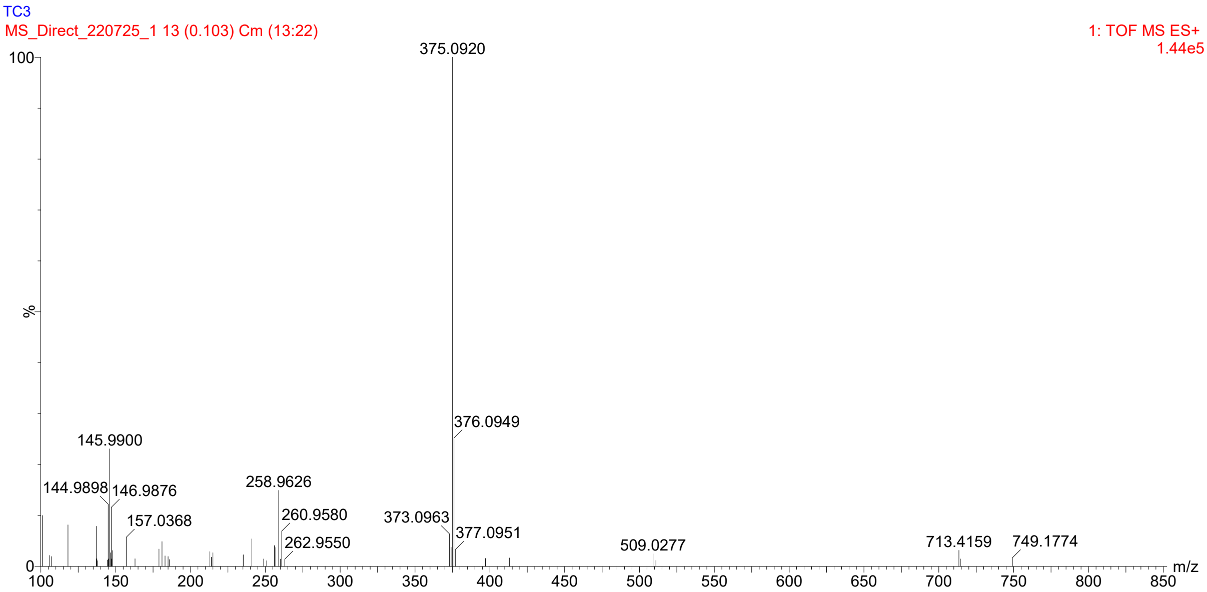

Figure S42: Experimental positive ionisation mode mass spectrum of **17**

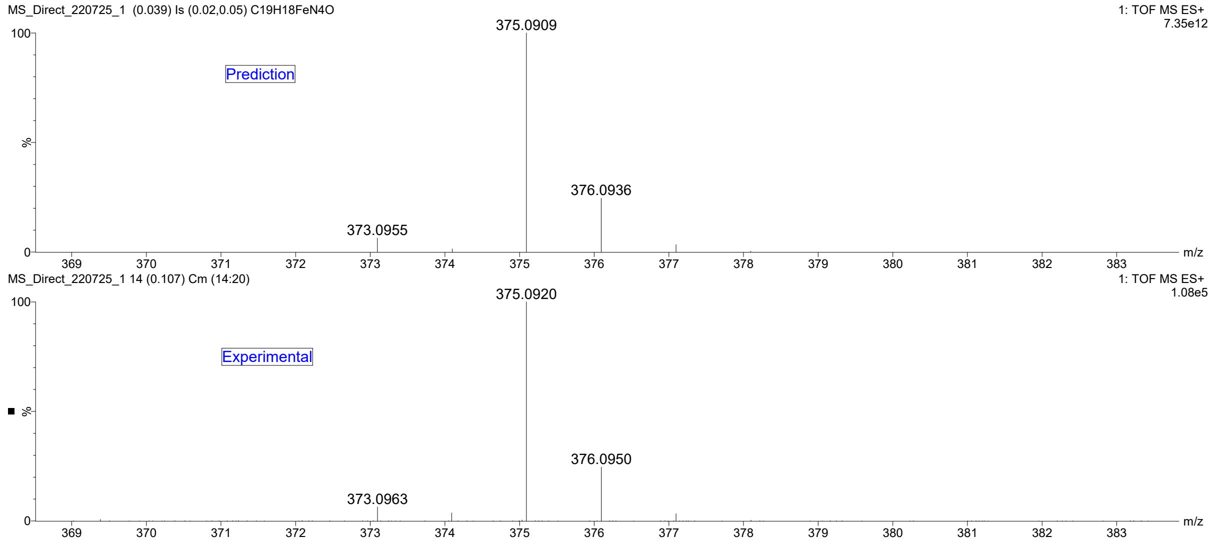

Figure 43: Predicted and experimental mass spectrum signal of **17**

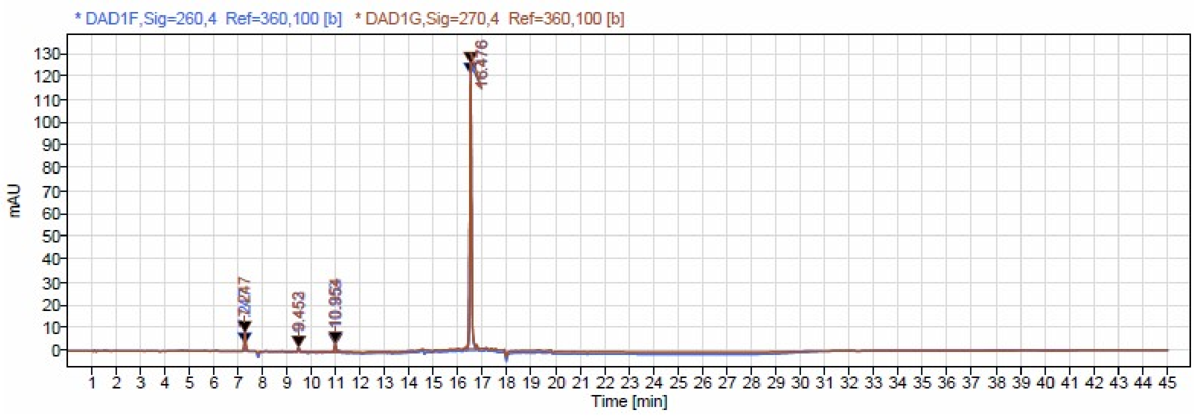

Signal: * DAD1F,Sig=260,4 Ref=360,100 [b]

RT [min]	Type	Width [min]	Area	Height	Area%	Name
7.247	MM m	0.15	11.75	2.66	1.90	
9.452	MM m	0.13	5.81	1.43	0.94	
10.953	MM m	0.11	13.00	2.83	2.10	
16.476	BV	0.37	588.04	120.94	95.06	
		**Sum**	**618.60**			

Figure S44: HPLC trace of **17**

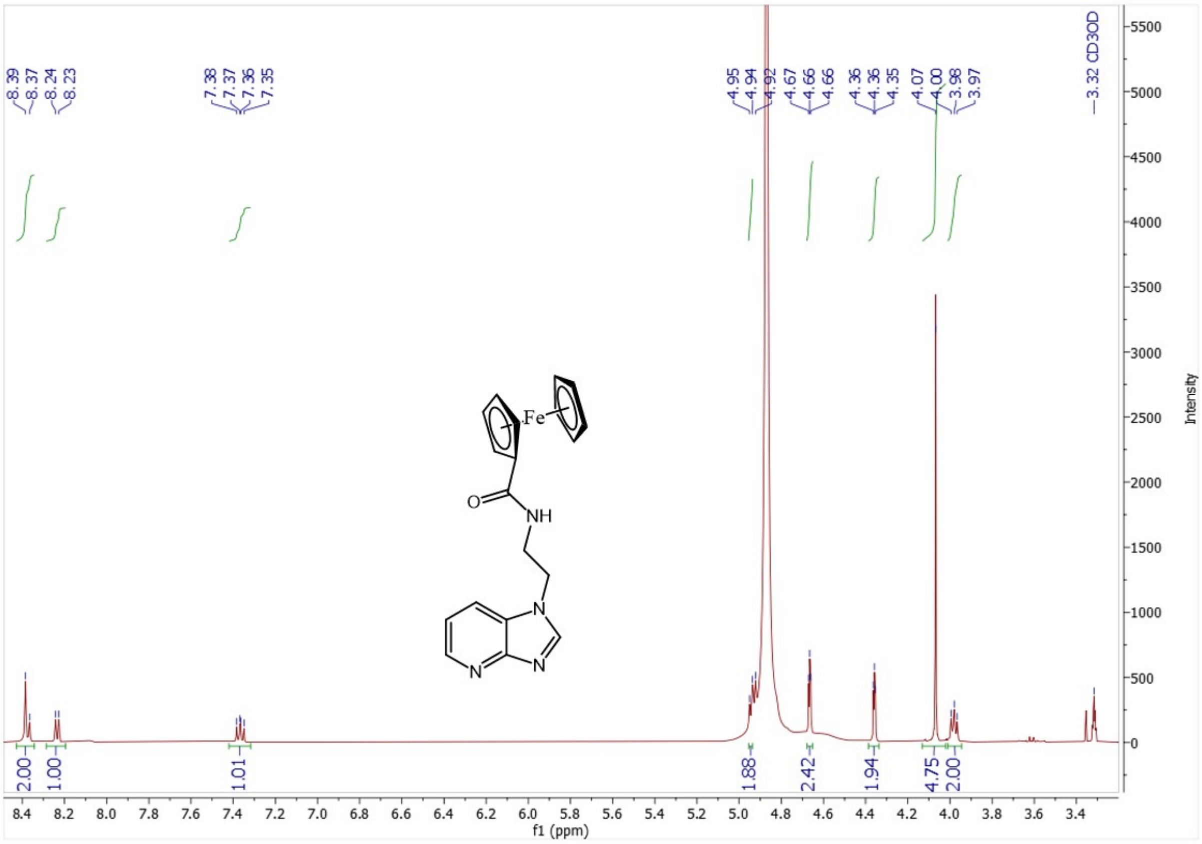

Figure S45: ^{1}H NMR spectrum of **16**

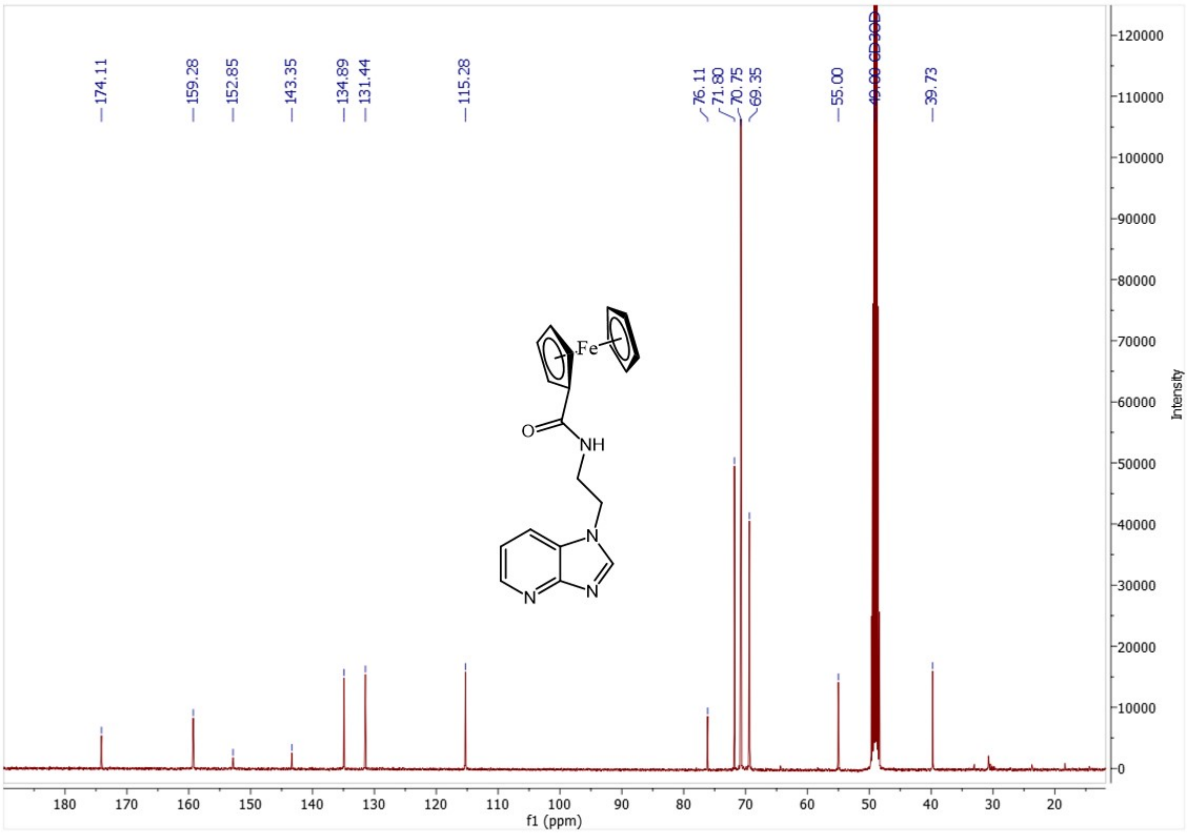

Figure S46: ^{13}C NMR spectrum of **16**

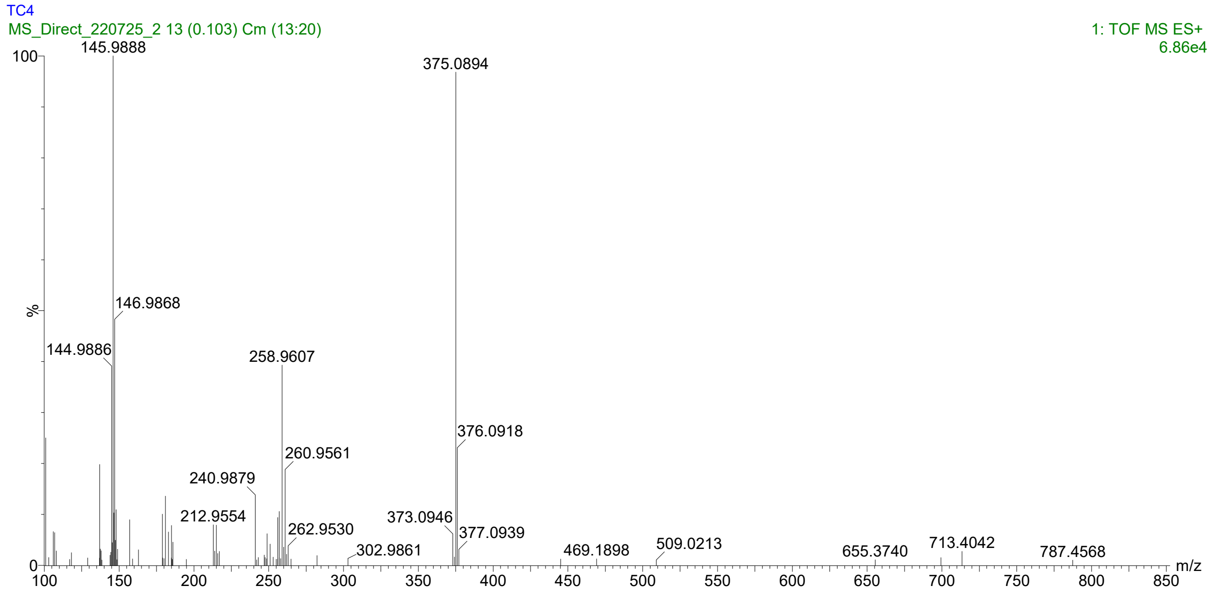

Figure S47: Experimental positive ionisation mode mass spectrum of **16**

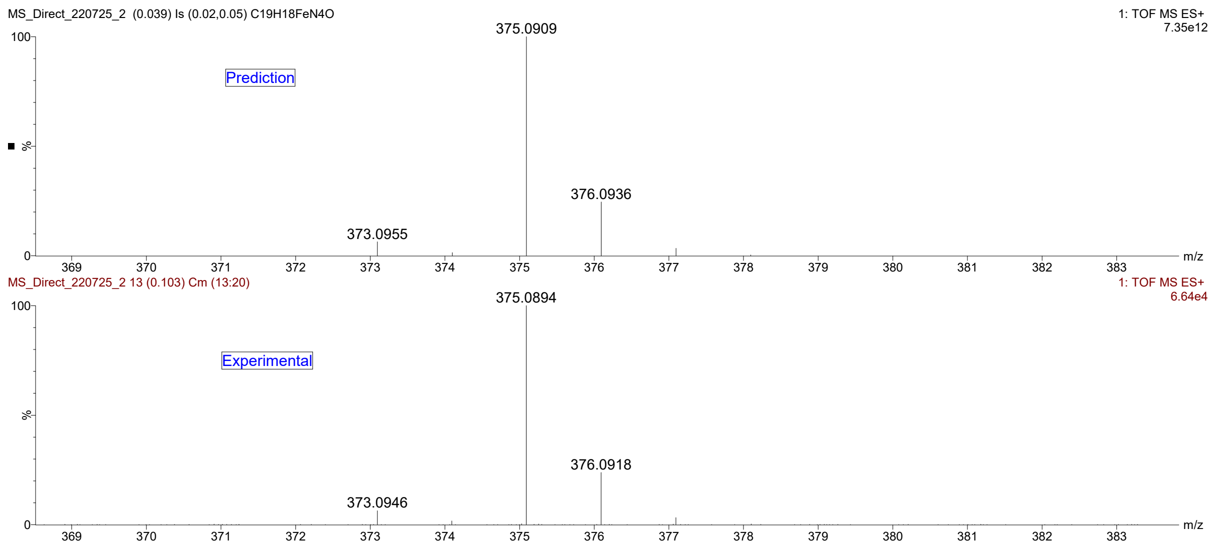

Figure S48: Predicted and experimental mass spectrum signal of **16**

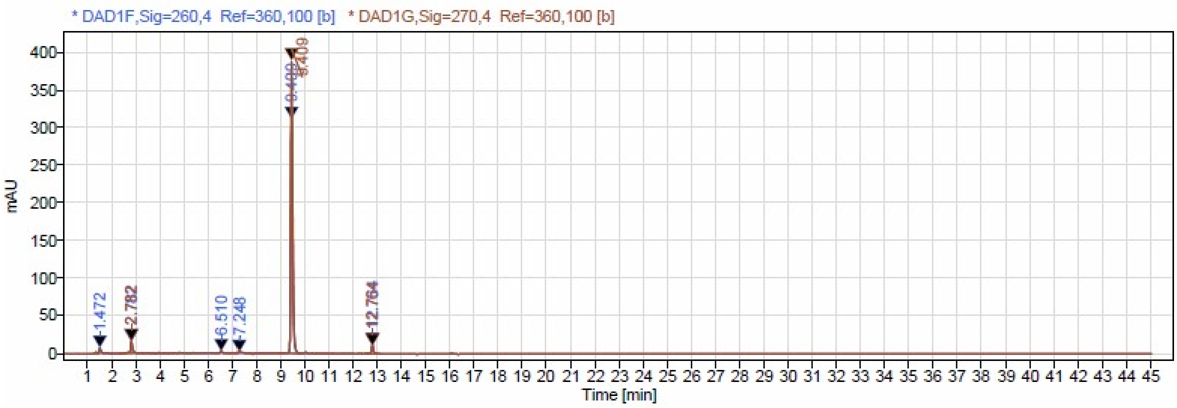

Signal: * DAD1F,Sig=260,4 Ref=360,100 [b]

RT [min]	Type	Width [min]	Area	Height	Area%	Name
1.472	MM m	0.24	36.73	7.17	1.93	
2.782	MM m	0.19	61.97	14.57	3.25	
6.510	MM m	0.14	8.03	2.18	0.42	
7.248	MM m	0.15	6.44	1.49	0.34	
9.409	BV	0.92	1723.93	311.45	90.53	
12.764	BB	0.75	67.10	12.08	3.52	
		**Sum**	**1904.19**			

Figure S49: HPLC trace of **16**

<u>Solubility studies</u>

Table S1. Solubility ranges of the target compounds in PBS and HEPES buffers at 25 and 30°C.

**Compound**	**PBS (pH7.4) 25 °C**		**PBS (pH7.4) 37 °C**		**HEPES (pH 7.0) 25 °C**		**HEPES (pH 7.0) 37 °C**	
	µM	µg/mL	µM	µg/mL	µM	µg/mL	µM	µg/mL
**14**	10-20	4-9	10-20	4-9	20-40	9-18	5-10	2-4
**15**	10-20	4-7	10-20	4-7	10-20	4-7	20-40	7-15
**16**	10-20	4-7	10-20	4-7	20-40	7-15	10-20	4-7
**17**	0-5	0-2	5-10	2-4	20-40	7-15	0-5	0-2
Reserpine	0-5	0-3	0-5	0-3	0-5	0-3	0-5	0-3
Hydrocortisone	>160	>60	>160	>60	>160	>60	>160	>60

Stability studies

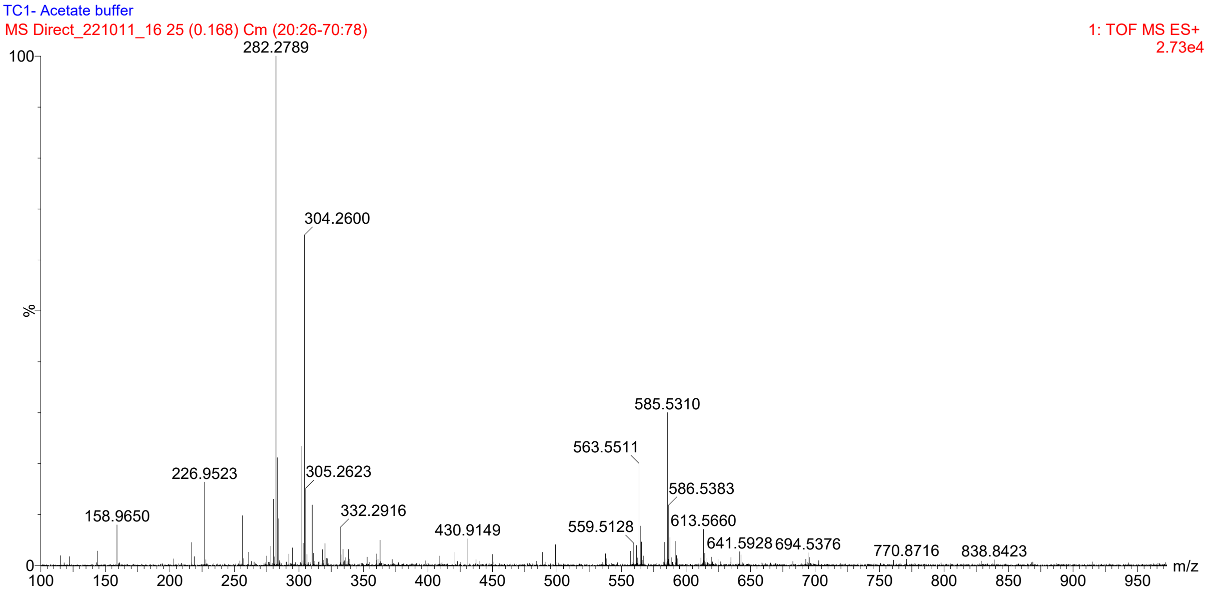

Figure S50: Experimental positive ionisation mode mass spectrum of **14** in acetate buffer.

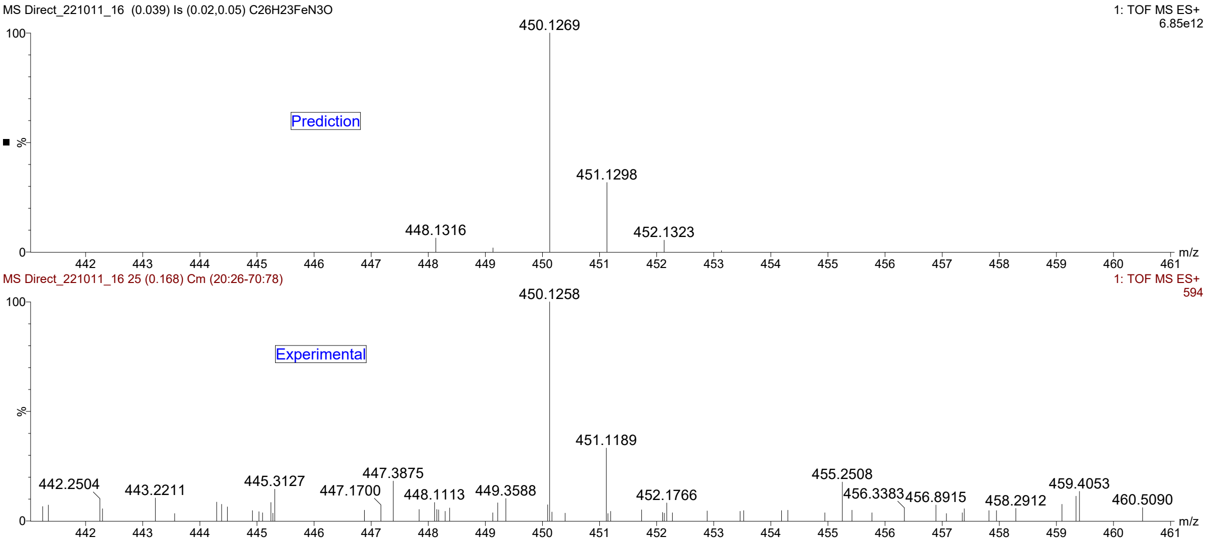

Figure S51: Predicted and experimental mass spectrum signal of **14** in acetate buffer.
